# Synthesis of D-*manno*-heptulose via a cascade aldol/hemiketalization reaction

**DOI:** 10.3762/bjoc.13.79

**Published:** 2017-04-28

**Authors:** Yan Chen, Xiaoman Wang, Junchang Wang, You Yang

**Affiliations:** 1 Shanghai Key Laboratory of New Drug Design, School of Pharmacy, East China University of Science and Technology, 130 Meilong Road, Shanghai 200237, China

**Keywords:** aldol reaction, cascade reaction, D-*manno*-heptulose, higher-carbon sugar, ketoheptose

## Abstract

A [4 + 3] synthesis of D-*manno*-heptulose is described. The cascade aldol/hemiketalization reaction of a C_4_ aldehyde with a C_3_ ketone provides the differentially protected ketoheptose building block, which can be further reacted to furnish target D-*manno*-heptulose.

## Introduction

D-*manno*-Heptulose is a rare naturally occurring seven-carbon sugar first isolated from avocado [1], which exhibited promising diabetogenic effects through suppression of the glucose metabolism and insulin secretion via competitive inhibition of the glucokinase pathway [2–6]. Accordingly, ketoheptoses and fluorinated ketoheptoses were considered to be potential therapeutic agents for hypoglycemia and cancer as well as diagnostic tools for diabetes [7–12]. Amino- and azido-group-containing ketoheptoses were also synthesized for the development of novel antibiotics and the evaluation of carbohydrate–lectin interactions by conjugation with fluorescent quantum dots via click chemistry [13–14]. Besides, differentially protected D-*manno*-heptulose building blocks could serve as valuable precursors for the synthesis of *C*-glycosides [15–16].

**Scheme 1:**
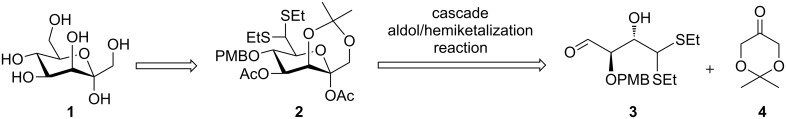
Retrosynthetic analysis of D-*manno*-heptulose.

The known synthesis of D-*manno*-heptulose mainly rely on the use of rearrangements and chain elongation reactions [17]. Rearrangement reactions such as the Lobry de Bruyn rearrangement and the Bilik rearrangement employ unprotected aldoses as substrates, usually yielding an equilibrium mixture of aldoses and ketoses [18–19]. In addition to chain elongations of aldoses employing the Henry reaction, the aldol reaction, and the Wittig reaction for the preparation of ketoheptoses [20–22], sugar lactones were also often utilized for the synthesis of D-*manno*-heptulose via reactions with *C*-nucleophiles or conversion into exocyclic glycals followed by dihydroxylation [10–13,23–27]. Remarkably, Thiem et al. reported the highly efficient synthesis of D-*manno*-heptulose from D-mannose in 59% overall yield over five steps [26]. However, the synthesis of D-*manno*-heptulose and its derivatives from the common differentially protected ketoheptose building block is still attractive due to the versatile functionalization possibilities of the building block into various derivatives of D-*manno*-heptulose. A de novo synthesis has proved to be an attractive strategy to produce orthogonally protected carbohydrate building blocks from simple precursors [28–39]. Here, we report a [4 + 3] approach to access differentially protected ketoheptose building blocks, which enables the synthesis of D-*manno*-heptulose. As depicted in Scheme 1, D-*manno*-heptulose (**1**) could be obtained by global deprotection of the differentially protected ketoheptose building block **2**. The ketoheptose **2** can be further divided into C_4_ aldehyde **3** and C_3_ ketone **4** via a cascade aldol/hemiketalization pathway.

## Results and Discussion

**Scheme 2:**
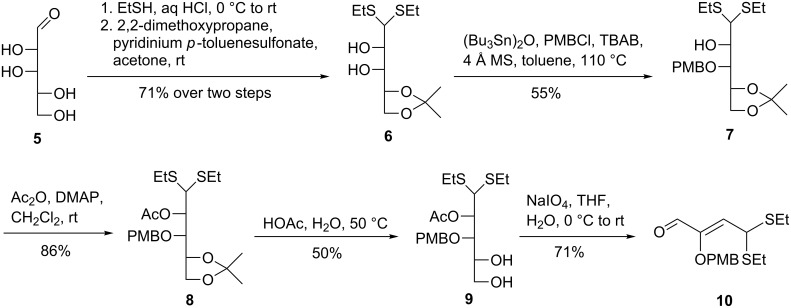
Initial attempt on the synthesis of the C_4_ aldehyde from D-lyxose (**5**).

The synthesis of the C_4_ aldehyde commenced with commercially available D-lyxose (**5**, Scheme 2). The reaction of **5** with ethanethiol in the presence of hydrochloric acid followed by selective protection of the 4,5-diol with 2,2-dimethoxypropane using pyridinium *p*-toluenesulfonate as the promoter gave the 4,5-*O*-isopropylidene derivative **6** in 71% yield over two steps [40]. Treatment of diol **6** with bis(tributyltin) oxide and subsequent exposure to *p*-methoxybenzyl (PMB) chloride in the presence of tetra-*n*-butylammonium bromide (TBAB) at 110 °C led to regioselective protection of the 3-OH with the PMB group, affording the 3-*O*-PMB protected alcohol **7** (55%) [41]. At this stage, we initially planned to synthesize the 2-OH-protected C_4_ aldehyde for the assembly of the seven-carbon skeleton. Thus, acetylation of the 2-OH group in **7** with acetic anhydride and DMAP in dichloromethane provided ester **8** in 86% yield. The positions of the 2-acetyl and 3-PMB groups were determined by ^1^H, ^13^C and 2D NMR spectra of **8** (see Supporting Information File 1 for details). Cleavage of the isopropylidene acetal group in **8** under acidic conditions gave diol **9** (50%). However, oxidative cleavage of diol **9** with sodium periodate resulted in the unexpected formation of α,β-unsaturated aldehyde **10** in 71% yield, indicating that the 2-acetyl group might be prone to initiate the elimination reaction. The double bond of **10** was assigned to have *Z*-configuration based on the analysis of the NOEs between the olefinic hydrogen and the aldehyde hydrogen (see Supporting Information File 1 for details). In addition, when alcohol **7** was subjected to benzoyl chloride and DMAP in dichloromethane at room temperature or *tert*-butyldimethylsilyl chloride and imidazole in DMF at room temperature, no reaction occurred probably because of the steric hindrance between the 2-OH group and the surrounding functional groups.

**Scheme 3:**
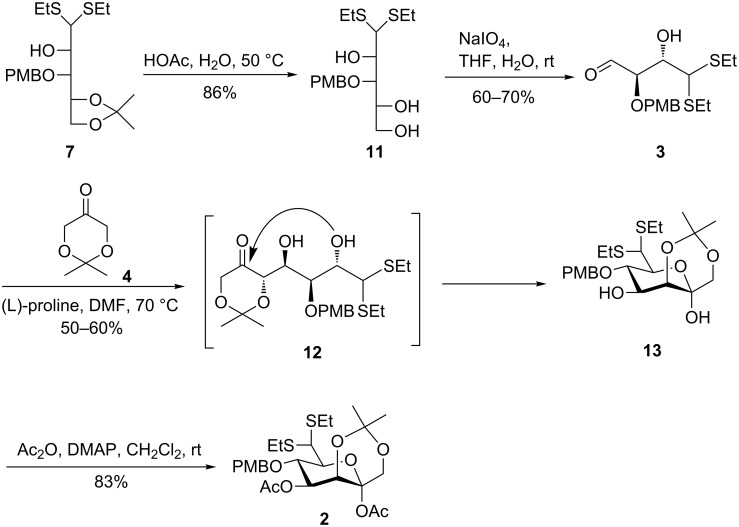
Synthesis of differentially protected ketoheptose building block **2**.

To overcome the difficulties in the synthesis of the 2-OH-protected C_4_ aldehyde and to improve the synthetic efficiency in the assembly of ketoheptose skeletons, we envisioned ketoheptoses could be assembled by a cascade aldol/hemiketalization reaction between 2-OH-unprotected C_4_ aldehyde **3** and C_3_ ketone **4**. As such, the isopropylidene acetal group in **7** was cleaved under acidic conditions to produce triol **11** in 86% yield (Scheme 3). Cleavage of the resulting vicinal diol in **11** with sodium periodate led to the C_4_ aldehyde **3** in nearly 60–70% yield. In this oxidative cleavage reaction, almost no elimination product was found based on TLC monitoring. Given that the C_4_ aldehyde **3** was unstable upon purification by silica gel column chromatography, it was immediately used for the subsequent coupling after the extraction procedure. The aldol reaction of aldehyde **3** with the readily available ketone **4** [42–43] under the catalysis of L-proline at room temperature for three days proceeded sluggishly, leading to the desired product in a very low yield. Gratifyingly, when the L-proline-catalyzed aldol reaction was performed at 70 °C for one day, the TLC indicated the complete consumption of aldehyde **3**, and the generated 4,5-*anti*-selective coupling intermediate **12** underwent in situ cyclization to provide hemiketal **13** as the major product in about 50–60% yield (35% overall yield from compound **11**). Notably, trace amounts of a stereoisomer and a minor highly polar unknown byproduct were also observed in this cascade reaction. The excellent *anti*-selectivity for the L-proline-catalyzed aldol reaction can be explained by the Houk–List transition state model [43–45]. Compound **13** was then acetylated to afford differentially protected ketoheptose building block **2** in 83% yield. The structure of **2** was unambiguously confirmed by ^1^H, ^13^C, and 2D NMR spectra (see Supporting Information File 1 for details). The anomeric α-configuration of compound **2** was confirmed by analysis of the NOE effects between the C-1 hydrogen and the C-5 hydrogen.

**Scheme 4:**
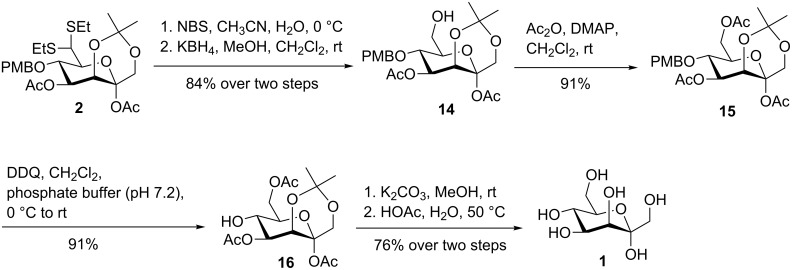
Synthesis of D-*manno*-heptulose (**1**).

With the ketoheptose building block **2** in hand, we turned our attention to the synthesis of D-*manno*-heptulose (**1**). Upon exposure to NBS in acetonitrile and water, the dithioacetal in **2** was cleaved to give the corresponding aldehyde [46–47], which was then reduced by potassium borohydride in a methanol and dichloromethane solvent mixture to produce alcohol **14** as the predominant product (84% over two steps, Scheme 4). In addition, a trace amount of the deacetylated product was also detected . DDQ-mediated oxidative cleavage of the PMB group in alcohol **14** produced only a moderate yield (≈50%) of the 5,7-diol probably due to the presence of the free 7-hydroxy group. We envisaged that protection of the free 7-hydroxy group in **14** followed by treatment with DDQ could yield the desired 5-hydroxy product in high yield. Indeed, acetylation of alcohol **14** with acetic anhydride delivered ester **15** in 91% yield. Removal of the PMB group in **15** with DDQ resulted in a very clean reaction, affording alcohol **16** in an excellent yield (91%). Saponification of all esters in **16** with potassium carbonate followed by acidic cleavage of the isopropylidene acetal group with aqueous acetic acid furnished D-*manno*-heptulose (**1**, 76% over two steps). The structure of **1** was found to be in good agreement with those reported for α-D-*manno*-heptulose (**1**) by comparison of the NMR spectra (see Supporting Information File 1 for details) [26].

## Conclusion

In summary, we have described a [4 + 3] approach for the synthesis of D-*manno*-heptulose (**1**) starting from D-lyxose (**5**). The key step is a cascade aldol/hemiketalization reaction for the construction of the differentially protected ketoheptose building block, which was finally converted into D-*manno*-heptulose for subsequent biological evaluation. Although the synthesis of D-*manno*-heptulose (5% overall yield, 13 steps) is not so efficient as the Thiem’s method (59% overall yield, 5 steps), the reported differentially protected ketoheptose building blocks may find further application in the preparation of structurally diverse D-*manno*-heptulose derivatives.

## Supporting Information

File 1 Experimental details, characterization data, and NMR spectra of all new compounds.

## References

[1] La Forge F B (1917). J Biol Chem.

[2] Roe J H, Hudson C S (1936). J Biol Chem.

[3] Simon E, Kraicer P F (1957). Arch Biochem Biophys.

[4] Paulsen E P, Richenderfer L, Winick P (1967). Nature.

[5] Coore H G, Randle P J (1964). Biochem J.

[6] Zelent D, Najafi H, Odili S, Buettger C, Weik-Collins H, Li C, Doliba N, Grimsby J, Matschinsky F M (2005). Biochem Soc Trans.

[7] Paulsen E P (1968). Ann N Y Acad Sci.

[8] Board M, Colquhoun A, Newsholme E A (1995). Cancer Res.

[9] Malaisse W J (2001). Diabetologia.

[10] Leshch Y, Waschke D, Thimm J, Thiem J (2011). Synthesis.

[11] Waschke D, Leshch Y, Thimm J, Himmelreich U, Thiem J (2012). Eur J Org Chem.

[12] Malaisse W J, Zhang Y, Louchami K, Sharma S, Dresselaers T, Himmelreich U, Novotny G W, Mandrup-Poulsen T, Waschke D, Leshch Y (2012). Arch Biochem Biophys.

[13] Leshch Y, Jacobsen A, Thimm A, Thiem J (2013). Org Lett.

[14] Schmidtke C, Kreuziger A-M, Alpers D, Jacobsen A, Leshch Y, Eggers R, Kloust H, Tran H, Ostermann J, Schotten T (2013). Langmuir.

[15] Levy D E, Tang C (1995). The chemistry of C-glycosides.

[16] Du Y, Linhardt R J, Vlahov I R (1998). Tetrahedron.

[17] Jacobsen A, Thiem J (2014). Curr Org Chem.

[18] Montgomery E M, Hudson C S (1939). J Am Chem Soc.

[19] Hricoviniova Z, Hricovini M, Petrusoa M, Matulova M, Petrus L (1998). Chem Pap.

[20] Sowden J C (1950). J Am Chem Soc.

[21] Schaffner R, Isbell H S (1962). J Org Chem.

[22] Cheng J, Fang Z, Li S, Zheng B, Jiang Y (2009). Carbohydr Res.

[23] Kampf A, Dimant E (1974). Carbohydr Res.

[24] Bessières B, Morin C (2003). J Org Chem.

[25] Liu X, Yin Q, Yin J, Chen G, Wang X, You Q-D, Chen Y-L, Xiong B, Shen J (2014). Eur J Org Chem.

[26] Waschke D, Thimm J, Thiem J (2011). Org Lett.

[27] Li X, Takahashi H, Ohtake H, Shiro M, Ikegami S (2001). Tetrahedron.

[28] Northrup A B, MacMillan D W C (2004). Science.

[29] Timmer M S M, Adibekian A, Seeberger P H (2005). Angew Chem, Int Ed.

[30] Ahmed M M, Berry B P, Hunter T J, Tomcik D J, O’Doherty G A (2005). Org Lett.

[31] Adibekian A, Timmer M S M, Stallforth P, van Rijn J, Werz D B, Seeberger P H (2008). Chem Commun.

[32] Stallforth P, Adibekian A, Seeberger P H (2008). Org Lett.

[33] Shan M, Xing Y, O’Doherty G A (2009). J Org Chem.

[34] Ohara T, Adibekian A, Esposito D, Stallforth P, Seeberger P H (2010). Chem Commun.

[35] Calin O, Pragani R, Seeberger P H (2012). J Org Chem.

[36] Babu R S, Chen Q, Kang S-W, Zhou M, O’Doherty G A (2012). J Am Chem Soc.

[37] Gati W, Rammah M M, Rammah M B, Couty F, Evano G (2012). J Am Chem Soc.

[38] Mlynarski J, Gut B (2012). Chem Soc Rev.

[39] Wang H-Y, Yang K, Yin D, Liu C, Glazier D A, Tang W (2015). Org Lett.

[40] van Delft F L, Rob A, Valentijn P M, van der Marel G A, van Boom J H (1999). J Carbohydr Chem.

[41] Grindley T B (1998). Adv Carbohydr Chem Biochem.

[42] Suri J T, Mitsumori S, Albertshofer K, Tanaka F, Barbas C F (2006). J Org Chem.

[43] Grondal C, Enders D (2006). Tetrahedron.

[44] Bahmanyar S, Houk K N, Martin H J, List B (2003). J Am Chem Soc.

[45] Hoang L, Bahmanyar S, Houk K N, List B (2003). J Am Chem Soc.

[46] Corey E J, Erickson B W (1971). J Org Chem.

[47] Crich D, de la Mora M A, Cruz R (2002). Tetrahedron.

