# Supplementary material for: Synthesis of D-manno-heptulose via a cascade aldol/hemiketalization reaction
Source: Beilstein J Org Chem. 2017 Apr 28;13:795–9. doi: 10.3762/bjoc.13.79 (PMC5433184; doi:10.3762/bjoc.13.79)
Supplement: File 1 — Experimental details, characterization data, and NMR spectra of all new compounds. [file Beilstein_J_Org_Chem-13-795-s001.pdf]

Supporting Information

for

Synthesis of D-*manno*-heptulose via a cascade aldol/hemiketalization reaction

Yan Chen, Xiaoman Wang, Junchang Wang, and You Yang*

Address: Shanghai Key Laboratory of New Drug Design, School of Pharmacy, East China University of Science and Technology, 130 Meilong Road, Shanghai 200237, China

Email: You Yang - yangyou@ecust.edu.cn

* Corresponding author

Experimental details, characterization data, and NMR spectra of all new compounds

Contents

	page
1. General information	S2
2. Experimental details and characterization data of new compounds	S2
3. References	S12
4. NMR spectra of new compounds	S13

1. General information. Commercial reagents were used without further purification except where noted. Solvents were dried and redistilled prior to use in the usual way. All reactions were performed in oven-dried glassware with magnetic stirring under an inert atmosphere unless noted otherwise. Analytical thin-layer chromatography (TLC) was performed on precoated plates of Silica Gel (0.25–0.3 mm, Shanghai, China). The TLC plates were visualized with UV light and by staining with a solution of ammonium molybdate and ammonium ceric nitrate in aqueous sulfuric acid or sulfuric acid-ethanol solution. Silica gel column chromatography was performed on Silica Gel AR (100–200 mesh, Shanghai, China). Optical rotations (OR) were measured with a Rudolph Research Analytical Autopol I automatic polarimeter at a concentration (*c*) expressed in g/100 mL. NMR spectra were measured with a Bruker Avance III 400 or Bruker Avance III 500 spectrometer. The ^{1}H and ^{13}C NMR spectra were calibrated against the residual proton and carbon signals of the solvents as internal references ($CDCl_3$: δ_H = 7.26 ppm and δ_C = 77.2 ppm; D_2O: δ_H = 4.79). Multiplicities are quoted as singlet (s), broad singlet (br s), doublet (d), doublet of doublets (dd), doublet of doublet of doublets (ddd), triplet (t), doublet of triplets (dt), quartet (q) or multiplet (m). Spectra were assigned using COSY, HSQC, and NOESY. All NMR chemical shifts (δ) were recorded in ppm and coupling constants (*J*) were reported in Hz. Mass spectra were recorded on an Agilent Technologies 6120 or LCT Premier XE FTMS instrument.

2. Experimental details and characterization data of new compounds

2.1. Synthesis of 4,5-*O*-isopropylidene-D-lyxose diethyl dithioacetal (6)

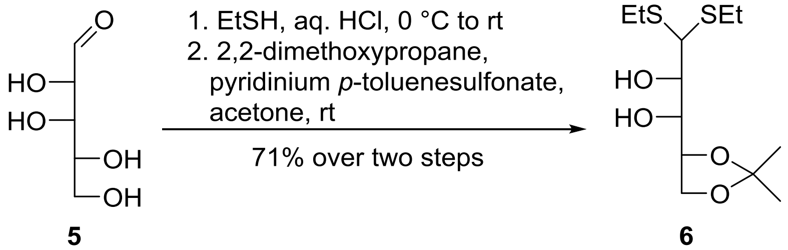

5 **6**

To a solution of D-lyxose (**5**, 8 g, 53.3 mmol) in concentrated aqueous HCl (8 mL, 37%) at 0 °C, was slowly added ethanethiol (8 mL). The reaction mixture was

warmed to room temperature and stirred for further 2 h. The mixture was neutralized by addition of aq ammonia upon which the white solid crystalized. The suspension was filtered and the residue was washed with hexane, dried by suction to obtain the corresponding dithioacetal as a white powder for the next step without further purification. LRMS (ESI) *m/z* calcd for $C_{10}H_{21}O_6S_2$ $[M + HCOO]^-$ 301.1, found 301.1. To a solution of the above dithioacetal and pyridinium *p*-toluenesulfonate (5.7 g, 22.7 mmol) in acetone (200 mL) at room temperature, was slowly added 2,2-dimethoxypropane (11.1 mL, 90.6 mmol) under argon [1]. After stirring at room temperature for 2 h, the reaction mixture was quenched with sat. aq $NaHCO_3$ and extracted with EtOAc. The organic layer was dried over Na_2SO_4, filtered, and concentrated in vacuo. The residue was purified by silica gel column chromatography (petroleum ether/EtOAc: 5/1) to give **6** (11.2 g, 71% over two steps) as a white solid: $[\alpha]^{20}_D = +19.6$ (*c* 2.0, $CHCl_3$); ^1H NMR (400 MHz, $CDCl_3$) δ 4.41 (dt, *J* = 3.2, 6.8 Hz, 1 H), 4.27 (d, *J* = 2.0 Hz, 1 H, H-1), 4.10 (dd, *J* = 6.8, 8.0 Hz, 1 H), 3.93 (t, *J* = 8.0 Hz, 1 H), 3.78 (dd, *J* = 2.4, 8.8 Hz, 1 H), 3.74 (dd, *J* = 3.2, 8.8 Hz, 1 H), 2.73 (q, *J* = 7.2 Hz, 2 H), 2.65 (q, *J* = 7.2 Hz, 2 H), 1.44 (s, 3 H), 1.38 (s, 3 H), 1.29 (t, *J* = 7.2 Hz, 3 H), 1.28 (t, *J* = 7.2 Hz, 3 H); ^{13}C NMR (100 MHz, $CDCl_3$) δ 109.4, 75.9, 74.1, 71.2, 66.6, 54.8, 26.6, 26.1, 25.8, 25.4, 14.8, 14.7; HRMS (ESI) *m/z* calcd for $C_{12}H_{24}O_4S_2Na$ $[M + Na]^+$ 319.1014, found 319.1011.

2.2. Synthesis of 3-*O*-*p*-methoxybenzyl-4,5-*O*-isopropylidene-D-lyxose diethyl dithioacetal (7)

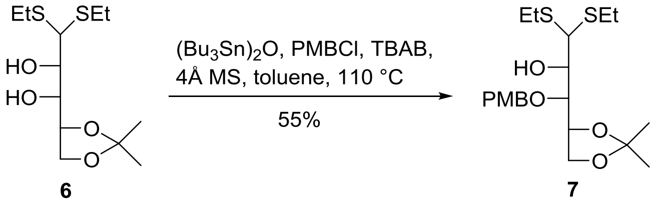

Analogous to a procedure described in [2] a mixture of compound **6** (1.15 g, 3.87 mmol), bis(tributyltin) oxide (2.85 mL, 5.84 mmol) and 4 Å MS (1.2 g) in toluene (42 mL) was heated at 110 °C for 4 h [3]. After cooling to room temperature, *p*-methoxybenzyl (PMB) chloride (1.05 mL, 7.73 mmol) and tetrabutylammonium

bromide (0.75 g, 2.32 mmol) were added, and the mixture was heated overnight at 110 °C. After cooling the mixture was filtered and the filtrate was evaporated. The residue was dissolved in CH_2Cl_2 and washed with water. The organic layer was dried over Na_2SO_4, filtered and concentrated in vacuo to give a residue, which was purified by silica gel column chromatography (petroleum ether/EtOAc: 8/1) to afford the 3-*O*-PMB protected alcohol **7** (0.89 g, 55%) as a pale yellow syrup. $[\alpha]^{20}_{D}$ = +26.6 (*c* 1.5, $CHCl_3$); ^{1}H NMR (400 MHz, $CDCl_3$) δ 7.27 (d, *J* = 8.8 Hz, 2 H, Ar), 6.87 (d, *J* = 8.8 Hz, 2 H, Ar), 4.79 (ABq, *J* = 10.8 Hz, 1 H, OCH_2Ar), 4.64 (ABq, *J* = 11.2 Hz, 1 H, OCH_2Ar), 4.36 (dd, *J* = 6.4, 13.6 Hz, 1 H), 4.15 (d, *J* = 2.8 Hz, 1 H, H-1), 4.06 (dd, *J* = 6.4, 8.4 Hz, 1 H), 3.91 (dt, *J* = 2.8, 8.0 Hz, 1 H), 3.88–3.81 (m, 2 H), 3.80 (s, 3 H), 2.98 (d, *J* = 8.0 Hz, 1 H, O*H*), 2.72–2.57 (m, 4 H), 1.45 (s, 3 H), 1.38 (s, 3 H), 1.24 (q, *J* = 7.2 Hz, 6 H); ^{13}C NMR (100 MHz, $CDCl_3$) δ 159.4, 130.7, 129.7, 113.9, 109.0, 79.0, 77.9, 74.0, 73.9, 66.6, 55.4, 54.7, 26.6, 25.7, 25.6, 25.5, 14.7, 14.6; HRMS (ESI) *m/z* calcd for $C_{20}H_{32}O_5S_2Na$ $[M + Na]^+$ 439.1589, found 439.1588.

2.3. Synthesis of 2-*O*-acetyl-3-*O*-*p*-methoxybenzyl-4,5-*O*-isopropylidene-D-lyxose diethyl dithioacetal (8)

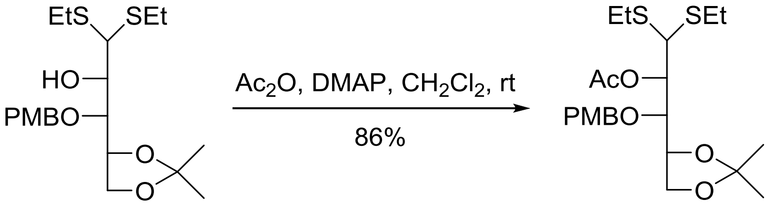

To a solution of alcohol **7** (1.68 g, 4.04 mmol) and DMAP (0.79 g, 6.48 mmol) in CH_2Cl_2 (50 mL) at room temperature, was added acetic anhydride (19 mL) under argon. After stirring at room temperature for 3 h, the mixture was washed with sat. aq $NaHCO_3$ and brine. The organic layer was dried over Na_2SO_4, filtered, and concentrated in vacuo. The residue was purified by silica gel column chromatography (petroleum ether/EtOAc: 15/1) to give **8** (1.58 g, 86%) as a pale yellow syrup: $[\alpha]^{20}_{D}$ = +1.3 (*c* 2.0, $CHCl_3$); ^{1}H NMR (400 MHz, $CDCl_3$) δ 7.28 (d, *J* = 8.8 Hz, 2 H, Ar), 6.87 (d, *J* = 8.8 Hz, 2 H, Ar), 5.27 (dd, *J* = 3.6, 8.0 Hz, 1 H, H-2), 4.85 (ABq, *J* = 10.8 Hz, 1 H, OCH_2Ar), 4.65 (ABq, *J* = 11.2 Hz, 1 H, OCH_2Ar), 4.21 (dd, *J* = 7.2, 13.6 Hz,

1 H, H-4), 4.13 (d, J = 3.2 Hz, 1 H, H-1), 3.94 (t, J = 7.6 Hz, 1 H, H-3), 3.89 (dd, J = 6.4, 8.0 Hz, 1 H, H-5a), 3.80 (s, 3 H), 3.76 (t, J = 8.0 Hz, 1 H, H-5b), 2.60 (m, 4 H), 2.09 (s, 3 H), 1.44 (s, 3 H), 1.35 (s, 3 H), 1.21 (dt, J = 7.6, 12.8 Hz, 6 H); ^{13}C NMR (100 MHz, $CDCl_3$) δ 169.9, 159.4, 130.5, 129.8, 113.9, 109.0, 79.1, 77.7, 74.3, 74.0, 66.5, 55.4, 51.6, 26.6, 25.8, 25.6, 25.5, 21.0, 14.5, 14.4; HRMS (ESI) *m/z* calcd for $C_{22}H_{34}O_6S_2Na$ [M + Na]$^+$ 481.1694, found 481.1689.

2.4. Synthesis of 2-*O*-acetyl-3-*O*-*p*-methoxybenzyl-D-lyxose diethyl dithioacetal (9)

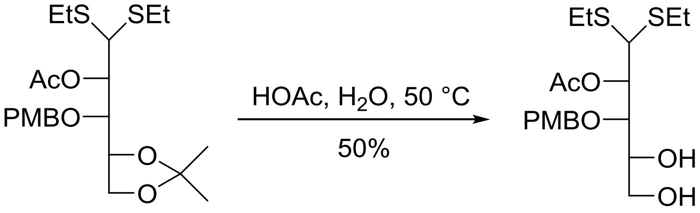

8 9

A solution of compound **8** (1.56 g, 3.41 mmol) in acetic acid/water (1/1, v/v, 30 mL) was stirred at 50 °C for 3 h. The mixture was concentrated in vacuo to give a residue, which was purified by silica gel column chromatography (CH_2Cl_2/MeOH: 120/1) to give **9** (0.71 g, 50%) as a pale yellow syrup: $[\alpha]^{20}_D$ = –27.7 (*c* 0.38, $CHCl_3$); ^{1}H NMR (400 MHz, $CDCl_3$) δ 7.27 (d, J = 8.0 Hz, 2 H, Ar), 6.88 (d, J = 8.4 Hz, 2 H, Ar), 5.43 (dd, J = 4.0, 7.2 Hz, 1 H, H-2), 4.72 (ABq, J = 11.2 Hz, 1 H, OCH_2Ar), 4.55 (ABq, J = 11.2 Hz, 1 H, OCH_2Ar), 4.14 (d, J = 4.0 Hz, 1 H, H-1), 3.93 (d-like, J = 7.6 Hz, 1 H), 3.80 (s, 3 H), 3.67 (m, 2 H), 3.54 (dd, J = 2.8, 9.6 Hz, 1 H), 2.73 (q, J = 7.6 Hz, 2 H), 2.62 (q, J = 7.6 Hz, 2 H), 2.15 (s, 3 H), 1.25 (m, 6 H); ^{13}C NMR (100 MHz, $CDCl_3$) δ 170.0, 159.7, 130.0, 129.6, 114.1, 76.9, 73.9, 73.7, 71.0, 64.3, 55.5, 51.7, 25.7, 25.3, 21.1, 14.5, 14.4; HRMS (ESI) *m/z* calcd for $C_{19}H_{30}O_6S_2Na$ [M + Na]$^+$ 441.1381, found 441.1377.

2.5. Synthesis of (*Z*)-4-(diethyl dithioacetal)-2-*O*-*p*-methoxybenzylbut-2-enal (10)

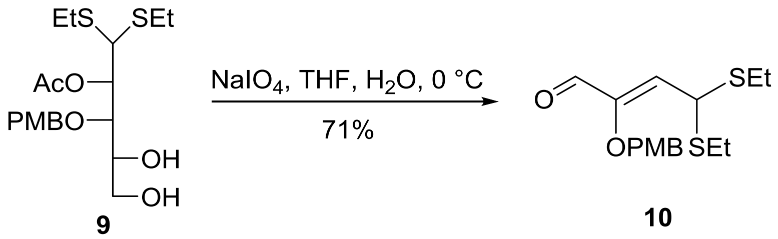

A solution of $NaIO_4$ (28 mg, 0.13 mmol) in water (0.36 mL) was added dropwise to a cooled (0 °C) and stirred solution of diol **9** (50 mg, 0.12 mmol) in THF (1.5 mL). The temperature was allowed to warm to room temperature and the stirring continued for 2 h. The mixture was diluted with hexane and washed with sat. aq $NaHCO_3$ and brine. The organic layer was dried over Na_2SO_4, filtered, and concentrated in vacuo. The residue was purified by silica gel column chromatography (petroleum ether/EtOAc: 10/1) to provide **10** (28 mg, 71%) as a yellow syrup: $[\alpha]^{20}_{D} = +7.0$ (*c* 0.4, $CHCl_3$); ^{1}H NMR (500 MHz, $CDCl_3$) δ 9.28 (s, 1 H, C*H*O), 7.27 (d, *J* = 8.0 Hz, 2 H, Ar), 6.87 (d, *J* = 8.5 Hz, 2 H, Ar), 5.88 (d, *J* = 10.5 Hz, 1 H), 5.07 (s, 2 H), 4.81 (d, *J* = 11.0 Hz, 1 H), 3.80 (s, 3 H), 2.53 (m, 4 H), 1.21 (t, *J* = 7.5 Hz, 6 H); ^{13}C NMR (100 MHz, $CDCl_3$) δ 189.1, 160.0, 151.9, 137.1, 130.4, 129.0, 114.1, 73.1, 55.5, 43.0, 25.7, 14.7; HRMS (ESI) *m/z* calcd for $C_{16}H_{22}O_3S_2Na$ $[M + Na]^+$ 349.0908, found 349.0902.

2.6. Synthesis of 3-*O*-*p*-methoxybenzyl-D-lyxose diethyl dithioacetal (11)

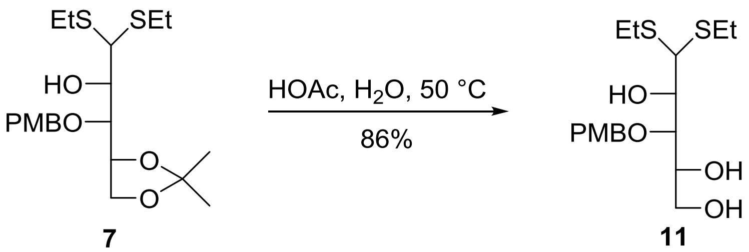

A solution of compound **7** (1.05 g, 2.5 mmol) in acetic acid/water (1/1, v/v, 5 mL) was stirred at 50 °C for 2 h. The mixture was concentrated in vacuo to give a residue, which was purified by silica gel column chromatography (CH_2Cl_2/MeOH: 40/1) to give **11** (0.82 g, 86%) as a pale yellow syrup: $[\alpha]^{20}_{D} = -9.3$ (*c* 2.05, $CHCl_3$); ^{1}H NMR (400 MHz, $CDCl_3$) δ 7.26 (d, *J* = 8.8 Hz, 2 H, Ar), 6.88 (d, *J* = 8.8 Hz, 2 H, Ar), 4.65 (ABq, *J* = 10.8 Hz, 1 H, OCH_2Ar), 4.57 (ABq, *J* = 10.8 Hz, 1 H, OCH_2Ar), 4.07

(d-like, *J* = 4.0 Hz, 1 H), 4.03 (m, 2 H), 3.90 (dd, *J* = 2.8, 7.2 Hz, 1 H), 3.80 (s, 3 H), 3.73 (dd, *J* = 6.8, 11.2 Hz, 1 H), 3.66 (dd, *J* = 4.4, 11.2 Hz, 1 H), 2.71 (m, 2 H), 2.61 (m, 2 H), 1.25 (q, *J* = 7.2 Hz, 6 H); ^{13}C NMR (100 MHz, $CDCl_3$) δ 159.7, 129.9, 129.8, 114.1, 77.9, 73.4, 73.3, 71.7, 64.2, 55.5, 54.9, 25.8, 25.3, 14.7, 14.6; HRMS (ESI) *m/z* calcd for $C_{17}H_{28}O_5S_2Na$ [M + Na]$^+$ 399.1276, found 399.1271.

2.7. Synthesis of 1,3-*O*-isopropylidene-5-*O*-*p*-methoxybenzyl-7-(diethyl dithioacetal)-α-D-*manno*-hept-2-ulopyranose (13)

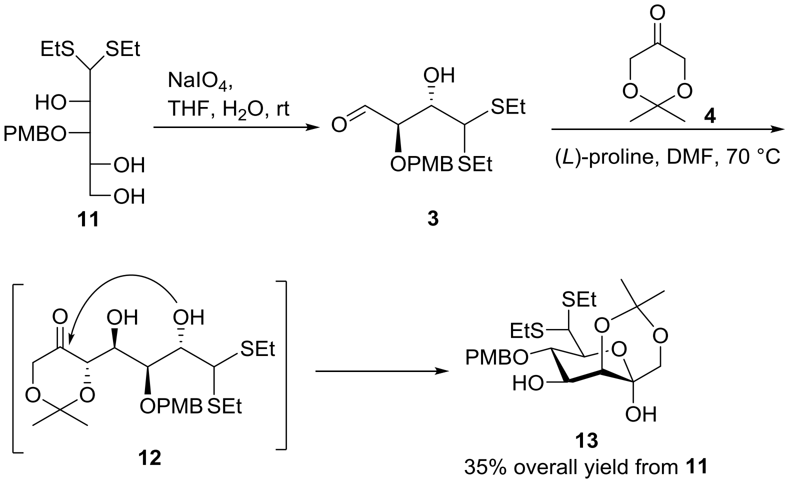

A solution of $NaIO_4$ (730 mg, 3.42 mmol) in water (1 mL) was added dropwise to a stirred solution of diol **11** (714 mg, 1.90 mmol) in THF (20 mL). The temperature was allowed to warm to room temperature and the stirring continued for 3 h. The mixture was diluted with dichloromethane and washed with water and saturated aqueous $NaHCO_3$. The organic layer was dried over Na_2SO_4, filtered, and concentrated in vacuo to give the corresponding aldehyde **3** as a yellow syrup for the next step without further purification. LRMS (ESI) *m/z* calcd for $C_{17}H_{25}O_6S_2$ [M + HCOO]$^-$ 389.1, found 389.1. To a suspension of dioxanone **4** [4,5] (494 mg, 3.79 mmol) and (L)-proline (65 mg, 0.57 mmol) in DMF (1 mL) at 70 °C, was added the above aldehyde **3**. After stirring at 70 °C for 1 day, the mixture was diluted with ethyl acetate and washed with water and brine, and dried over Na_2SO_4. Filtration, concentration in vacuo and elution through silica gel column chromatography (petroleum ether/EtOAc: 2/1) provided **13** (312 mg, 35% over two steps) as a pale yellow syrup: $[\alpha]^{20}_D$ = +4.3 (*c* 0.4, $CHCl_3$); ^{1}H NMR (500 MHz, $CDCl_3$) δ 7.28 (d, *J* =

8.5 Hz, 2 H, Ar), 6.86 (d, *J* = 8.5 Hz, 2 H, Ar), 4.94 (ABq, *J* = 10.5 Hz, 1 H, OC*H*$_2$Ar), 4.65 (ABq, *J* = 10.5 Hz, 1 H, OC*H*$_2$Ar), 4.17 (br s, 1 H), 4.11 – 4.07 (m, 3 H), 4.04 (m, 1 H), 3.79 (s, 3 H), 3.71 (ABq, *J* = 11.5 Hz, 1 H), 3.61 (ABq, *J* = 11.5 Hz, 1 H), 3.10 (d, *J* = 8.0 Hz, 1 H, O*H*), 2.90 (m, 1 H), 2.80 (m, 1 H), 2.68 (m, 2 H), 2.58 (br s, 1 H, O*H*), 1.45 (s, 3 H), 1.44 (s, 3 H), 1.24 (m, 6 H); ^{13}C NMR (125 MHz, CDCl$_3$) δ 159.1, 131.1, 129.7, 114.0, 99.5, 91.0, 77.7, 77.3, 75.0, 71.3, 71.2, 67.4, 55.4, 52.1, 28.5, 26.2, 24.3, 19.1, 14.6, 14.5; HRMS (ESI) *m/z* calcd for $C_{22}H_{34}O_7S_2Na$ [M + Na]$^+$ 497.1644, found 497.1646.

2.8. Synthesis of 1,3-*O*-isopropylidene-2,4-di-*O*-acetyl-5-*O*-*p*-methoxybenzyl-7-(diethyl dithioacetal)-α-D-*manno*-hept-2-ulopyranose (2)

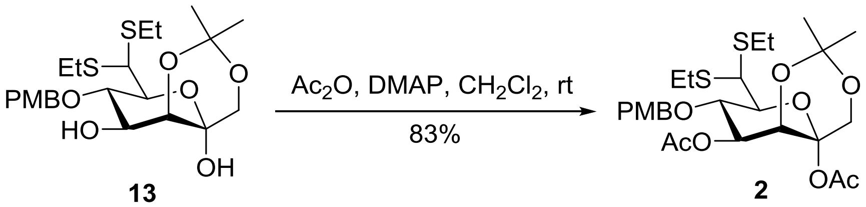

To a solution of diol **13** (140 mg, 0.30 mmol) and DMAP (130 mg, 1.06 mmol) in CH_2Cl_2 (3 mL) at room temperature, was added acetic anhydride (0.84 mL, 8.86 mmol) under argon. After stirring at room temperature for 4 h, the mixture was washed with sat. aq $NaHCO_3$ and brine. The organic layer was dried over Na_2SO_4, filtered, and concentrated in vacuo. The residue was purified by silica gel column chromatography (petroleum ether/EtOAc: 8/1) to afford **2** (137 mg, 83%) as a pale yellow syrup: $[\alpha]^{20}_D$ = –1.7 (*c* 1.03, CHCl$_3$); ^{1}H NMR (400 MHz, CDCl$_3$) δ 7.20 (d, *J* = 8.4 Hz, 2 H, Ar), 6.86 (d, *J* = 8.4 Hz, 2 H, Ar), 5.22 (dd, *J* = 3.2, 10.0 Hz, 1 H, H-4), 4.70 (ABq, *J* = 10.8 Hz, 2 H, OC*H*$_2$Ar), 4.47 (t, *J* = 9.6 Hz, 1 H, H-5), 4.39 (d, *J* = 3.2 Hz, 1 H, H-3), 4.29 (ABq, *J* = 12.4 Hz, 1 H, H-1a), 4.17 (ABq, *J* = 12.4 Hz, 1 H, H-1b), 4.16 (d, *J* = 1.6 Hz, 1 H, H-7), 4.01 (dd, *J* = 1.6, 9.6 Hz, 1 H, H-6), 3.80 (s, 3 H), 2.85 (m, 2 H), 2.73 (m, 2 H), 2.12 (s, 3 H), 2.08 (s, 3 H), 1.44 (s, 3 H), 1.41 (s, 3 H), 1.24 (m, 6 H); ^{13}C NMR (125 MHz, CDCl$_3$) δ 170.4, 168.2, 159.5, 130.5, 129.4, 114.1, 99.9, 98.3, 80.0, 75.2, 73.9, 72.8, 67.9, 65.2, 55.5, 51.5, 27.0, 25.4, 24.3, 21.6, 21.2, 20.6, 14.5, 14.4; HRMS (ESI) *m/z* calcd for $C_{26}H_{38}O_9S_2Na$ [M + Na]$^+$ 581.1855,

found 581.1849.

2.9. Synthesis of 1,3-*O*-isopropylidene-2,4-di-*O*-acetyl-5-*O*-*p*-methoxybenzyl-α-D-*manno*-hept-2-ulopyranose (14)

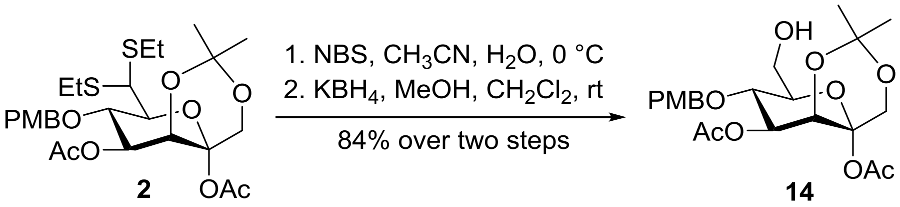

To a solution of dithioacetal **2** (717 mg, 1.28 mmol) in acetonitrile (46 mL) at 0 °C, was added a solution of NBS (1.37 g, 7.71 mmol) in acetonitrile/water (4/1, v/v, 27.5 mL) [6,7]. After stirring at 0 °C for 1 h, the mixture was diluted with EtOAc, washed with sat. aq $NaHCO_3$ and brine. The organic layer was dried over Na_2SO_4, filtered, and concentrated in vacuo to give the corresponding aldehyde as a pale yellow foam for the next step without further purification. To a solution of the above aldehyde in methanol/dichloromethane (1/1, v/v, 40 mL) at room temperature, was added KBH_4 (104 mg, 1.93 mmol). After stirring at room temperature for 1 h, the mixture was quenched with water (5 mL). Concentration in vacuo and elution through silica gel column chromatography (petroleum ether/EtOAc: 1/1) afforded **14** (492 mg, 84% over two steps) as a colorless syrup: $[\alpha]^{20}_D$ = +1.2 (*c* 1.2, $CHCl_3$); ^{1}H NMR (400 MHz, $CDCl_3$) δ 7.20 (d, *J* = 8.8 Hz, 2 H, Ar), 6.86 (d, *J* = 8.8 Hz, 2 H, Ar), 5.22 (dd, *J* = 3.6, 10.0 Hz, 1 H, H-4), 4.67 (ABq, *J* = 10.8 Hz, 1 H, OCH_2Ar), 4.57 (ABq, *J* = 10.4 Hz, 1 H, OCH_2Ar), 4.42 (d, *J* = 3.6 Hz, 1 H, H-3), 4.33 (ABq, *J* = 12.4 Hz, 1 H, H-1a), 4.13 (ABq, *J* = 12.8 Hz, 1 H, H-1b), 4.39 (d, *J* = 3.2 Hz, 1 H, H-3), 4.09 (t, *J* = 10.0 Hz, 1 H, H-5), 3.85 (dd, *J* = 2.4, 11.2 Hz, 1 H, H-7a), 3.79 (s, 3 H), 3.77 (m, 1 H, H-7b), 3.62 (ddd, *J* = 2.4, 3.6, 9.6 Hz, 1 H, H-6), 2.12 (s, 3 H), 2.06 (s, 3 H), 1.44 (s, 3 H), 1.42 (s, 3 H); ^{13}C NMR (100 MHz, $CDCl_3$) δ 170.5, 168.6, 159.6, 130.1, 129.6, 114.1, 100.0, 98.2, 75.2, 74.7, 72.4, 71.7, 68.1, 65.2, 61.7, 55.5, 27.4, 21.7, 21.3, 20.1; HRMS (ESI) *m/z* calcd for $C_{22}H_{30}O_{10}Na$ $[M + Na]^+$ 477.1737, found 477.1733.

2.10. Synthesis of 1,3-*O*-isopropylidene-2,4,7-tri-*O*-acetyl-5-*O*-*p*-methoxybenzyl-7-α-D-*manno*-hept-2-ulopyranose (15)

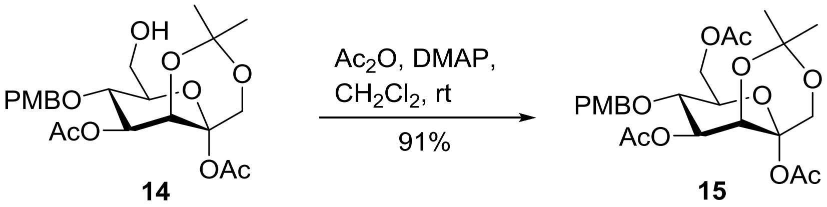

To a solution of alcohol **14** (59 mg, 0.13 mmol) and DMAP (32 mg, 0.26 mmol) in CH_2Cl_2 (5 mL) at room temperature, was added acetic anhydride (0.37 mL, 3.9 mmol) under argon. After stirring at room temperature for 4 h, the mixture was washed with sat. aq $NaHCO_3$ and brine. The organic layer was dried over Na_2SO_4, filtered, and concentrated in vacuo. The residue was purified by silica gel column chromatography (petroleum ether/EtOAc: 3/1) to give **15** (58 mg, 91%) as a colorless syrup: $[\alpha]^{20}_{D}$ = +8.8 (*c* 0.48, $CHCl_3$); ^{1}H NMR (400 MHz, $CDCl_3$) δ 7.20 (d, *J* = 8.8 Hz, 2 H, Ar), 6.86 (d, *J* = 8.4 Hz, 2 H, Ar), 5.21 (dd, *J* = 3.6, 10.0 Hz, 1 H, H-4), 4.65 (ABq, *J* = 10.4 Hz, 1 H, OCH_2Ar), 4.48 (ABq, *J* = 10.4 Hz, 1 H, OCH_2Ar), 4.44 (d, *J* = 3.6 Hz, 1 H, H-3), 4.32 (d-like, *J* = 3.2 Hz, 2 H), 4.27 (ABq, *J* = 12.4 Hz, 1 H, H-1a), 4.17 (ABq, *J* = 12.4 Hz, 1 H, H-1b), 3.98 (t, *J* = 10.0 Hz, 1 H, H-5), 3.79 (s, 3 H), 3.78 (m, 1 H), 2.14 (s, 3 H), 2.08 (s, 3 H), 2.07 (s, 3 H), 1.44 (s, 3 H), 1.41 (s, 3 H); ^{13}C NMR (100 MHz, $CDCl_3$) δ 171.0, 170.3, 168.4, 159.7, 129.8, 129.7, 114.1, 100.1, 98.6, 75.1, 72.6, 72.2, 71.8, 67.8, 65.2, 62.9, 55.5, 26.9, 21.7, 21.3, 21.0, 20.8; HRMS (ESI) *m/z* calcd for $C_{24}H_{32}O_{11}Na$ $[M + Na]^+$ 519.1842, found 519.1841.

2.11. Synthesis of 1,3-*O*-isopropylidene-2,4,7-tri-*O*-acetyl-α-D-*manno*-hept-2-ulopyranose (16)

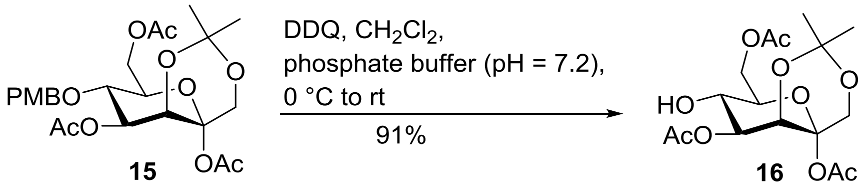

To a mixture of compound **15** (114 mg, 0.23 mmol) in dichloromethane (7.7 ml) and phosphate-buffer (30 mM, pH 7.2, 0.77 mL) at 0 °C, DDQ (156 mg, 0.69 mmol) was added portion-wise over 1 h. The reaction mixture was warmed to room

temperature and stirred for further 3 h. The mixture was diluted with sat. aq. $NaHCO_3$ solution, extracted with dichloromethane, and the organic layer was dried over Na_2SO_4 and concentrated in vacuo. The residue was purified by silica gel column chromatography (CH_2Cl_2/MeOH: 25/1) to give **16** (78 mg, 91%) as a colorless syrup: $[\alpha]^{20}_{D} = -31.8$ (*c* 0.6, $CHCl_3$); ^1H NMR (400 MHz, $CDCl_3$) δ 5.08 (dd, *J* = 3.6, 10.0 Hz, 1 H, H-4), 4.53 (dd, *J* = 4.0, 12.0 Hz, 1 H, H-7a), 4.39 (d, *J* = 3.6 Hz, 1 H, H-3), 4.28 (ABq, *J* = 12.8 Hz, 1 H, H-1a), 4.26 (dd, *J* = 2.4, 12.4 Hz, 1 H, H-7b), 4.16 (ABq, *J* = 12.4 Hz, 1 H, H-1b), 3.94 (t, *J* = 10.0 Hz, 1 H, H-5), 3.72 (ddd, *J* = 2.4, 4.0, 10.0 Hz, 1 H, H-6), 2.14 (s, 3 H), 2.11 (s, 3 H), 2.09 (s, 3 H), 1.40 (s, 6 H); ^{13}C NMR (100 MHz, $CDCl_3$) δ 172.1, 171.1, 168.4, 100.1, 98.7, 73.4, 71.7, 67.7, 65.2, 64.5, 63.0, 26.9, 21.7, 21.2, 21.0, 20.7; HRMS (ESI) *m/z* calcd for $C_{16}H_{24}O_{10}Na$ $[M + Na]^+$ 399.1267, found 399.1269.

2.12. Synthesis of α-D-*manno*-hept-2-ulopyranose (1)

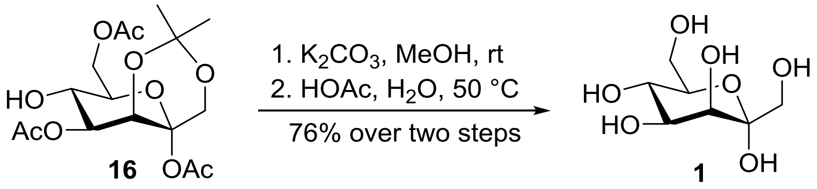

To a solution of compound **16** (68 mg, 0.18 mmol) in methanol (12 mL) at room temperature, was added potassium carbonate (38 mg, 0.27 mmol) at room temperature. After stirring at room temperature overnight, the reaction mixture was diluted with methanol and neutralized with Amberlite IR120 H^+ resin. After filtration, the filtrate was concentrated in vacuo to give the corresponding tetraol as a colorless syrup. A solution of the above tetraol in acetic acid/water (3/2, v/v, 5 mL) was stirred at 50 °C for overnight. Concentration in vacuo and elution through reverse phase C-18 column (H_2O) provided **1** [8] (29 mg, 76%) as a colorless syrup: $[\alpha]^{20}_{D} = +27.6$ (*c* 0.23, H_2O); ^1H NMR (400 MHz, D_2O) δ 3.93 (dd, *J* = 3.6, 9.6 Hz, 1 H, H-4), 3.90 (d, *J* = 3.6 Hz, 1 H, H-3), 3.87 (dd, *J* = 1.6, 11.6 Hz, 1 H, H-7a), 3.80 (ddd, *J* = 2.0, 6.0, 9.6 Hz, 1 H, H-6), 3.74 (dd, *J* = 6.4, 11.6 Hz, 1 H, H-7b), 3.72 (ABq, *J* = 11.6 Hz, 1 H, H-1a), 3.61 (t, *J* = 9.6 Hz, 1 H, H-5), 3.57 (ABq, *J* = 11.6 Hz, 1 H, H-1b); ^{13}C NMR (100 MHz,

D_2O) δ 97.7 (C-2), 73.0 (C-6), 70.9 (C-4), 69.9 (C-3), 66.9 (C-5), 64.0 (C-1), 61.0 (C-7); HRMS (ESI) *m/z* calcd for $C_7H_{13}O_7$ $[M – H]^-$ 209.0661, found 209.0668.

3. References

(1) van Delft, F. L.; Valentijn, A. R. P. M.; van der Marel, G. A.; van Boom, J. H. *J. Carbohydr. Chem.* **1999**, *18*, 165–190.

(2) Seeberger, P. H.; Yang, Y.; Anish, C.; Reinhardt, A. Total Synthesis and Immunological Evaluation of Saccharide Moieties of the Lipopolysaccharide from *Neisseria meningitidis*. WO 2013/041732 A1, March 28, 2013.

(3) Grindley, T. B. *Adv. Carbohydr. Chem. Biochem.* **1998**, *53*, 17–142.

(4) Suri, J. T.; Mitsumori, S.; Albertshofer, K.; Tanaka, F.; Barbas III, C. F. *J. Org. Chem.* **2006**, *71*, 3822–3828.

(5) Grondal, C.; Enders, D. *Tetrahedron* **2006**, *62*, 329–337.

(6) Corey, E. J.; Erickson, B. W. *J. Org. Chem.* **1971**, *36*, 3553–3560.

(7) Crich, D.; de la Mora, M. A.; Cruz, R. *Tetrahedron* **2002**, *58*, 35–44.

(8) Waschke, D.; Thimm, J.; Thiem, J. *Org. Lett.* **2011**, *13*, 3628–3631.

CDCl$_3$, 400 MHz

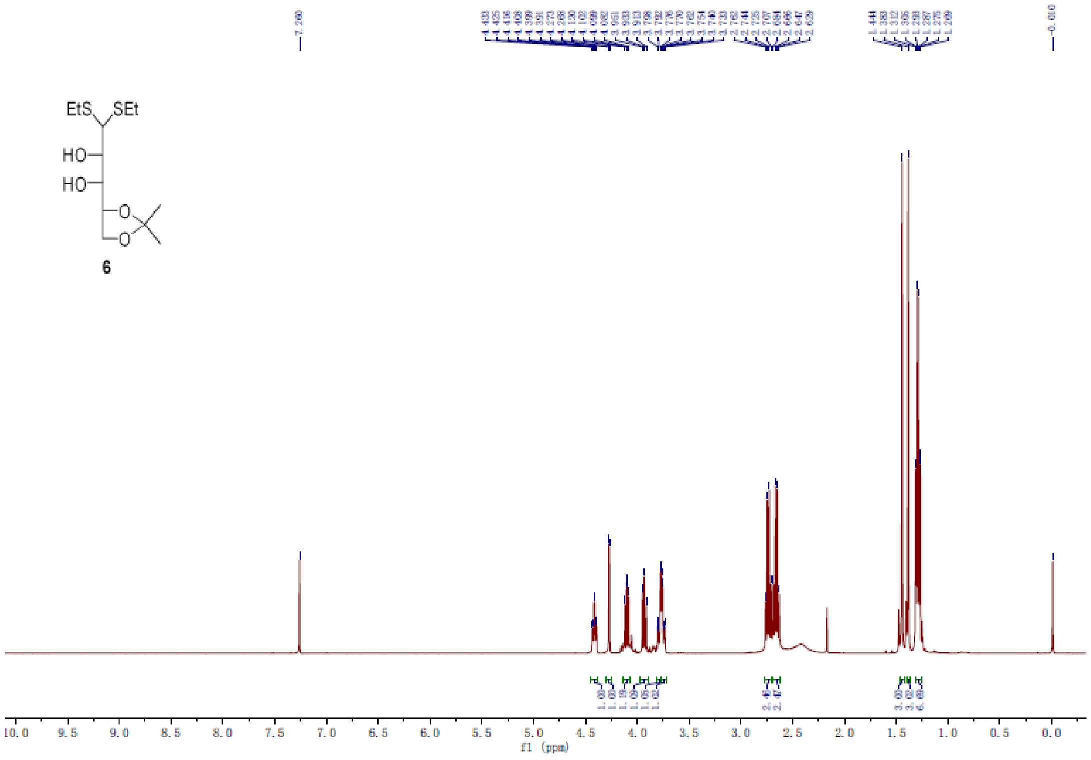

CDCl$_3$, 100 MHz

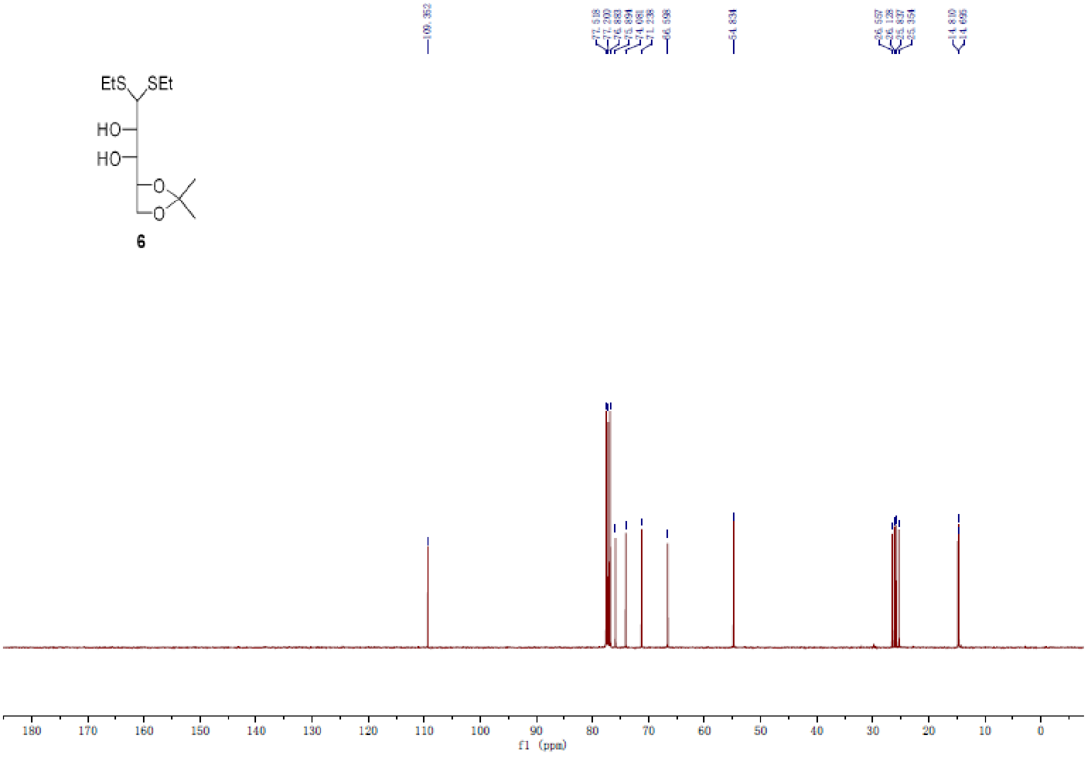

CDCl$_3$, 400 MHz

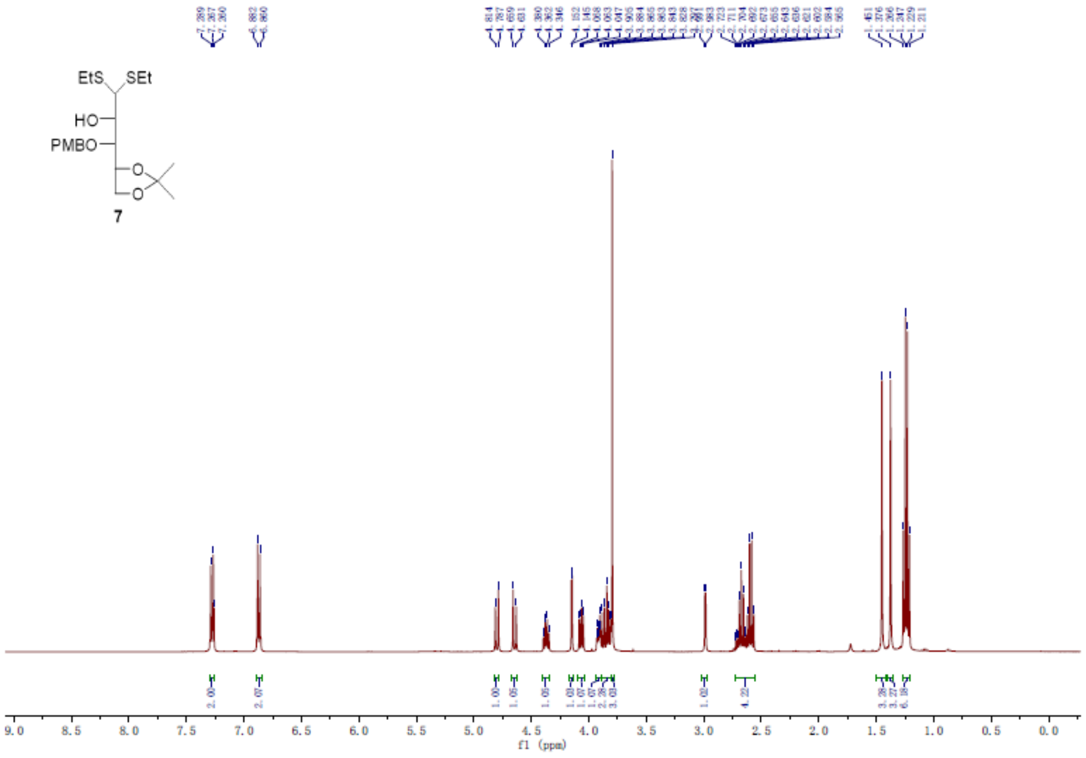

CDCl$_3$, 100 MHz

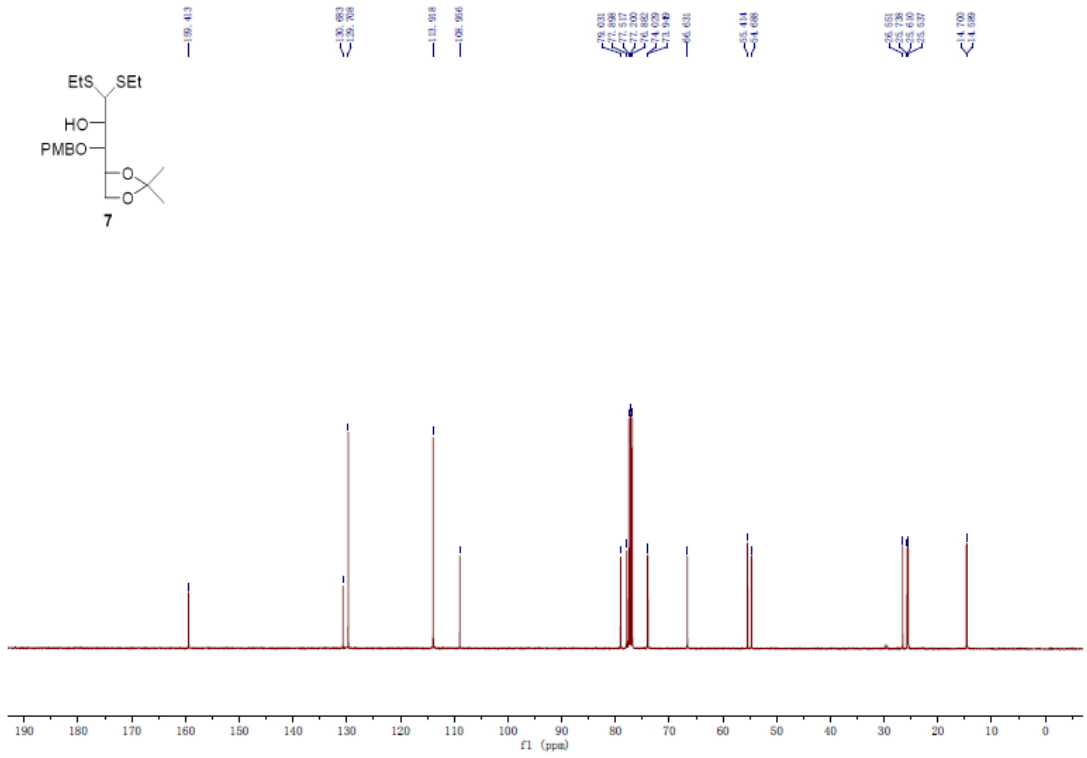

CDCl$_3$, 400 MHz

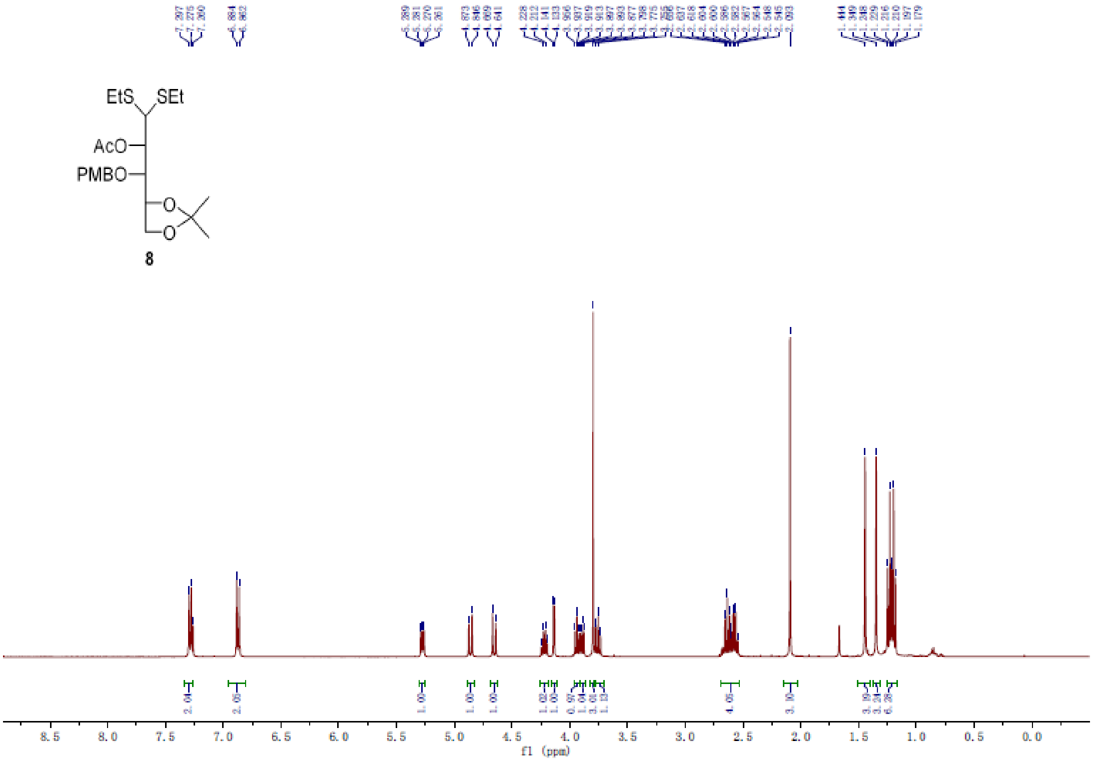

CDCl$_3$, 100 MHz

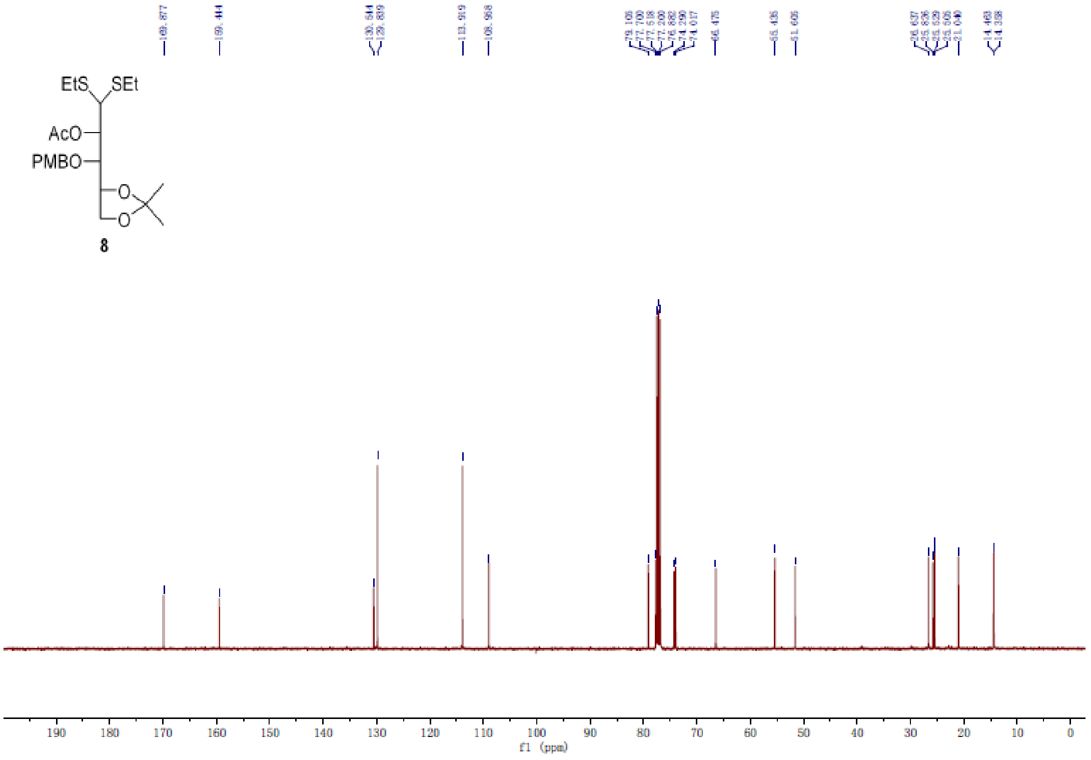

CDCl$_3$, 400 MHz

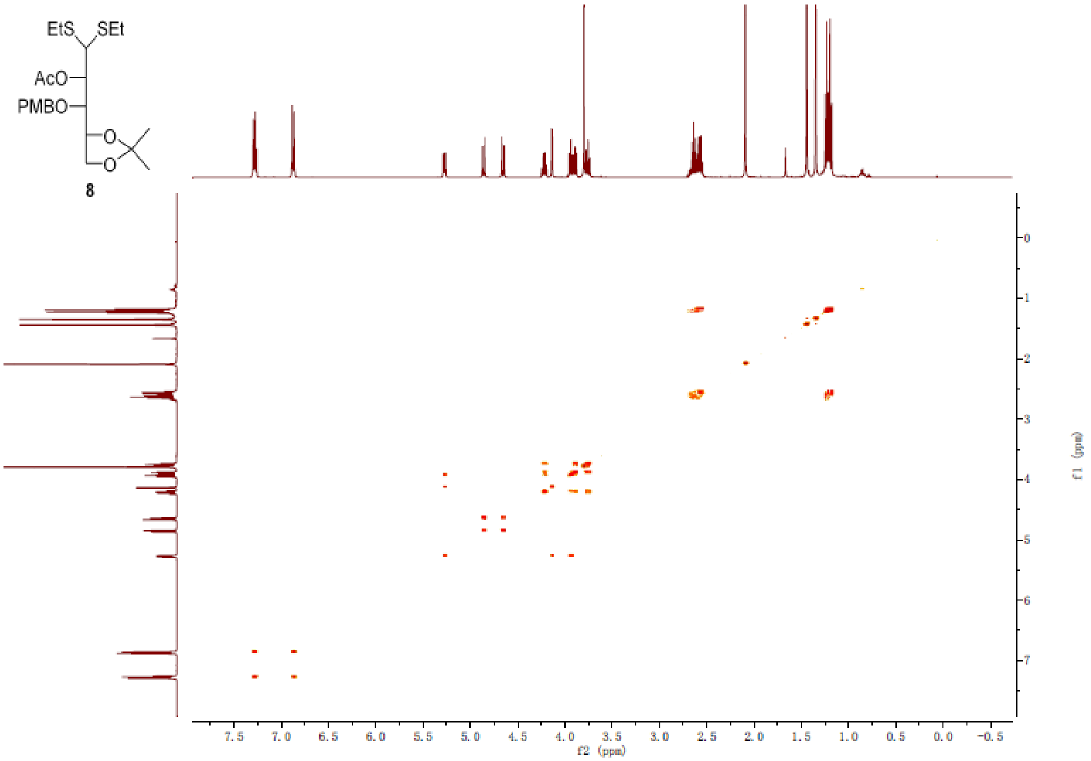

CDCl$_3$, 400 MHz

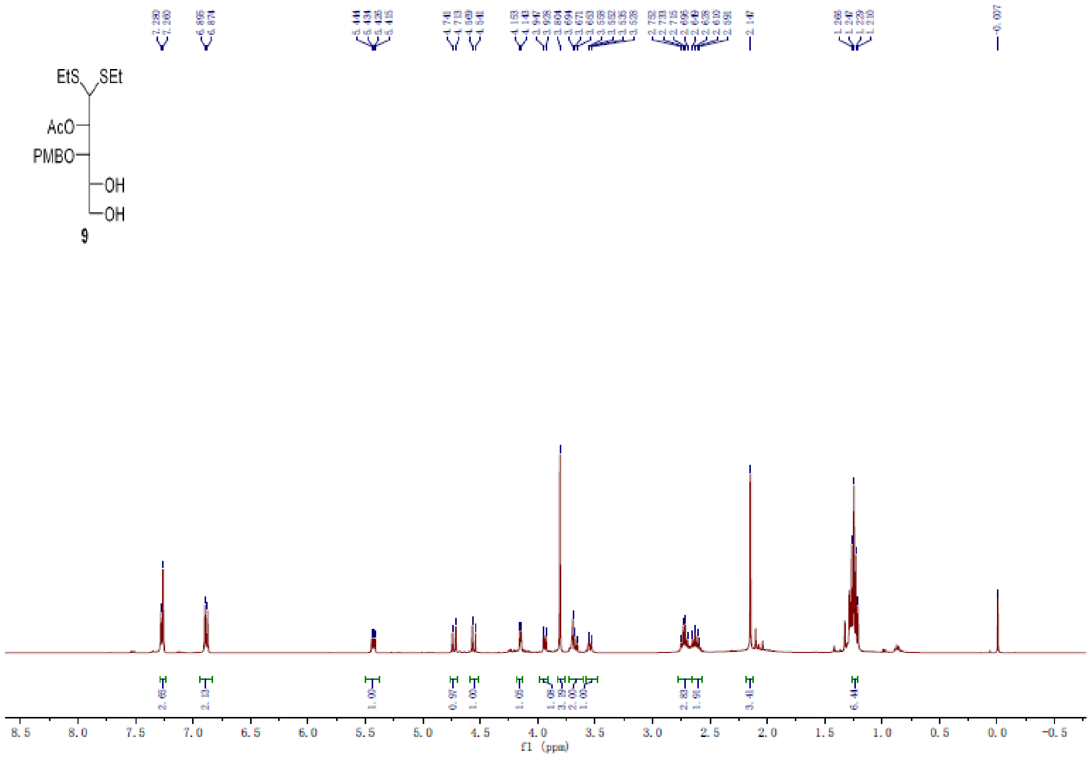

CDCl$_3$, 100 MHz

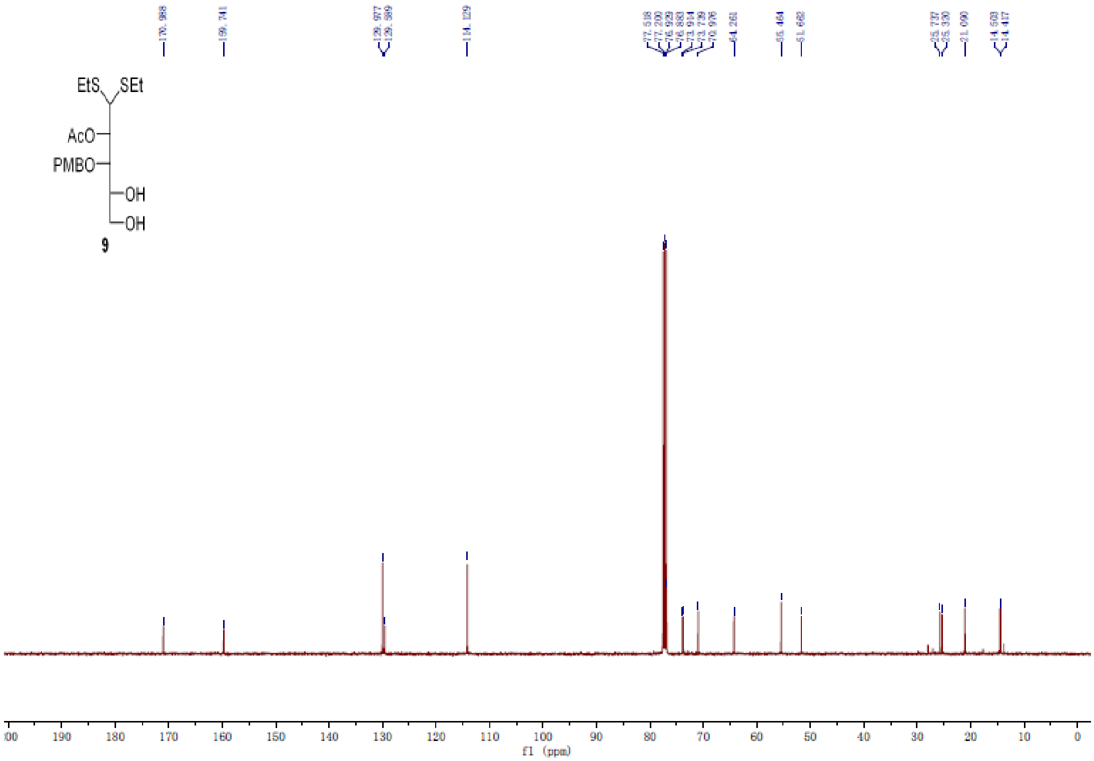

CDCl$_3$, 500 MHz

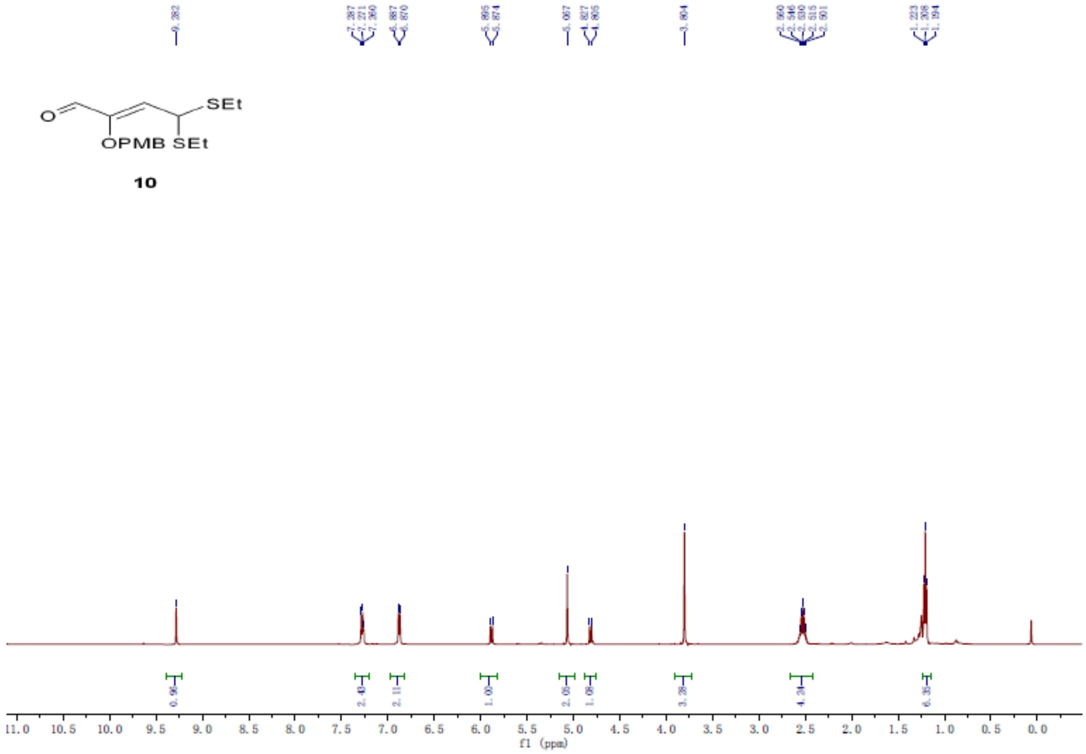

CDCl$_3$, 100 MHz

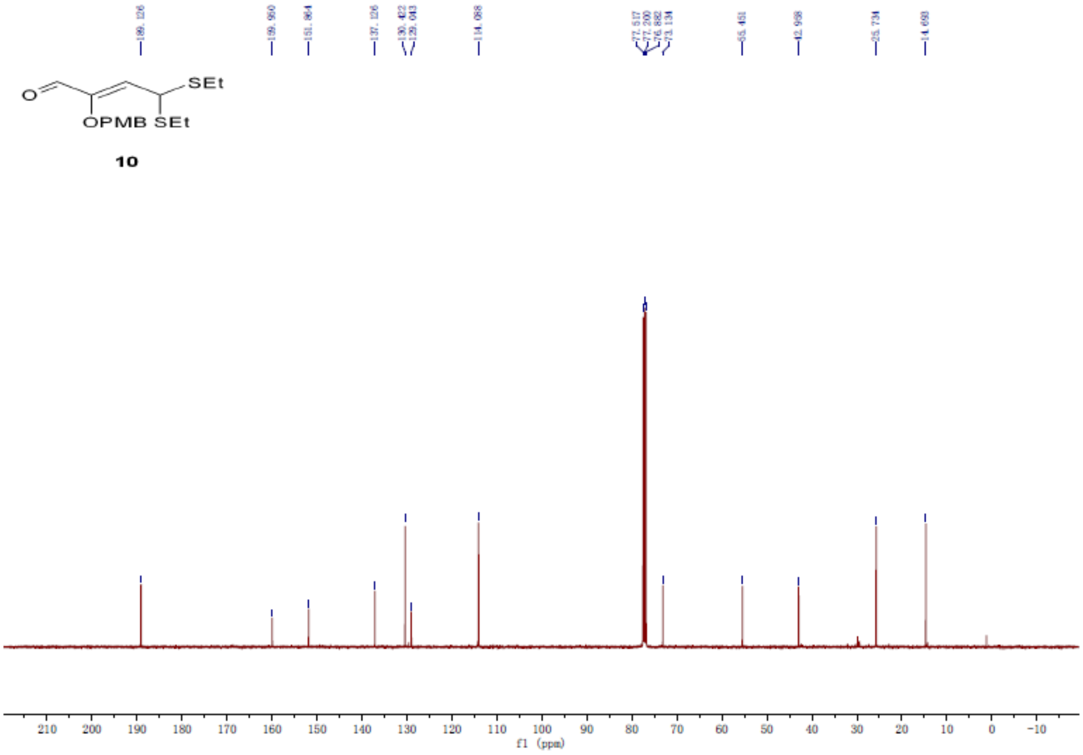

CDCl$_3$, 500 MHz

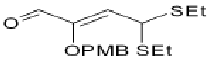

10

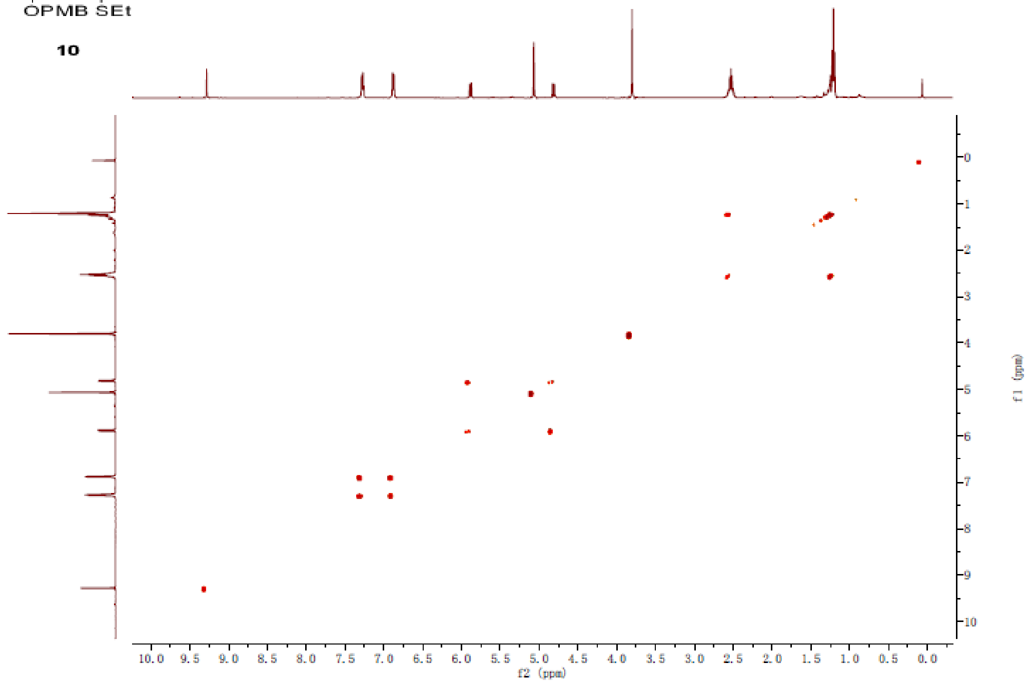

$CDCl_3$, 500 MHz

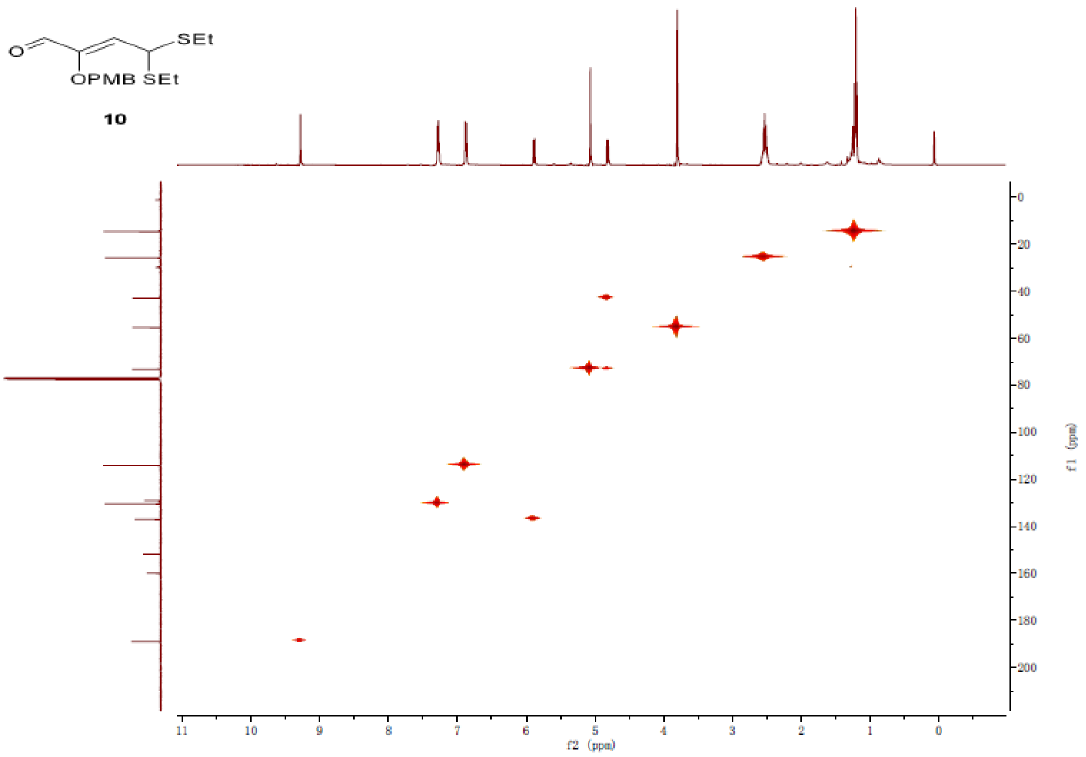

$CDCl_3$, 500 MHz

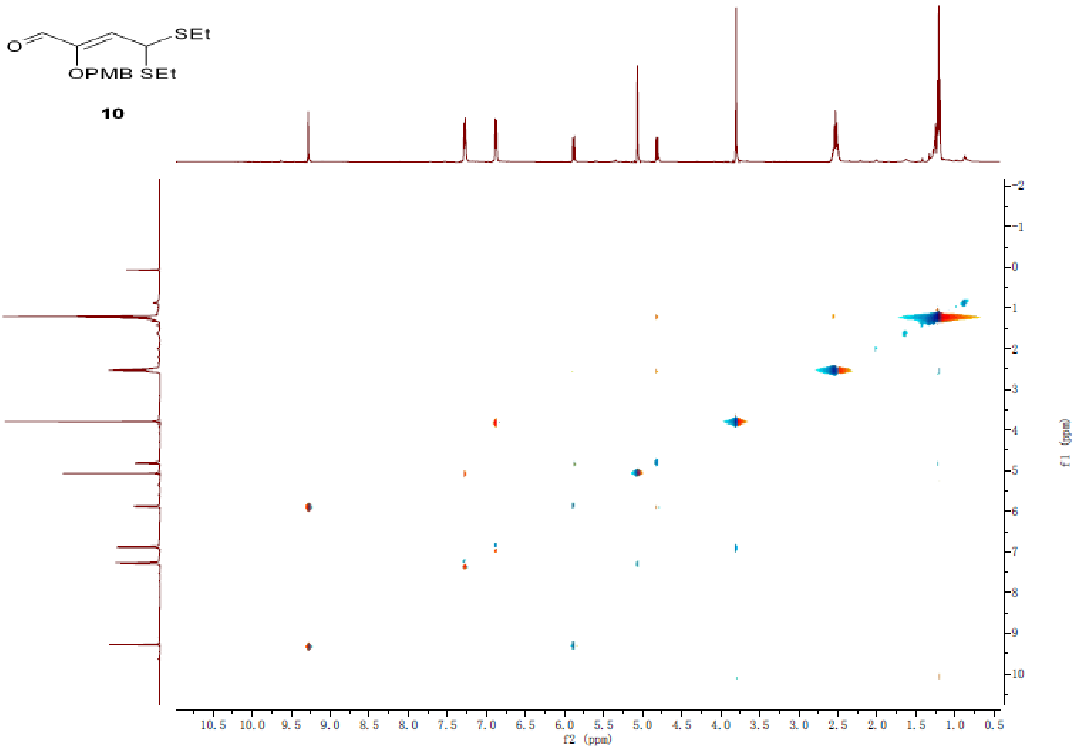

CDCl$_3$, 400 MHz

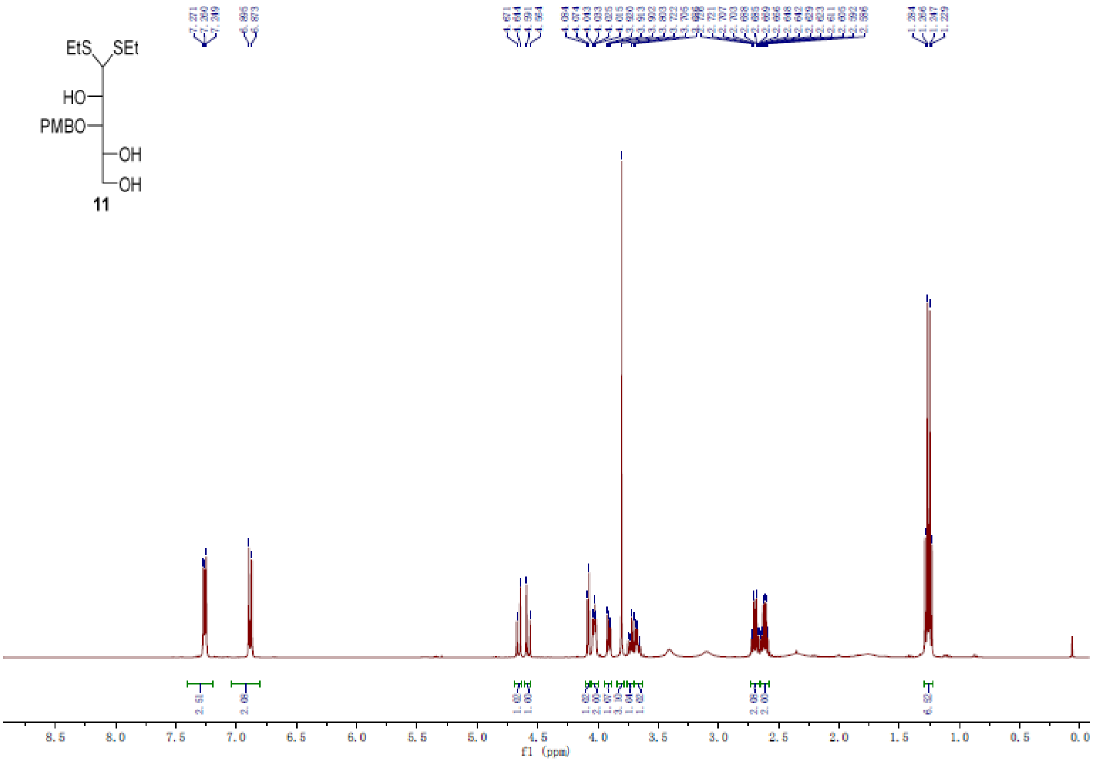

CDCl$_3$, 100 MHz

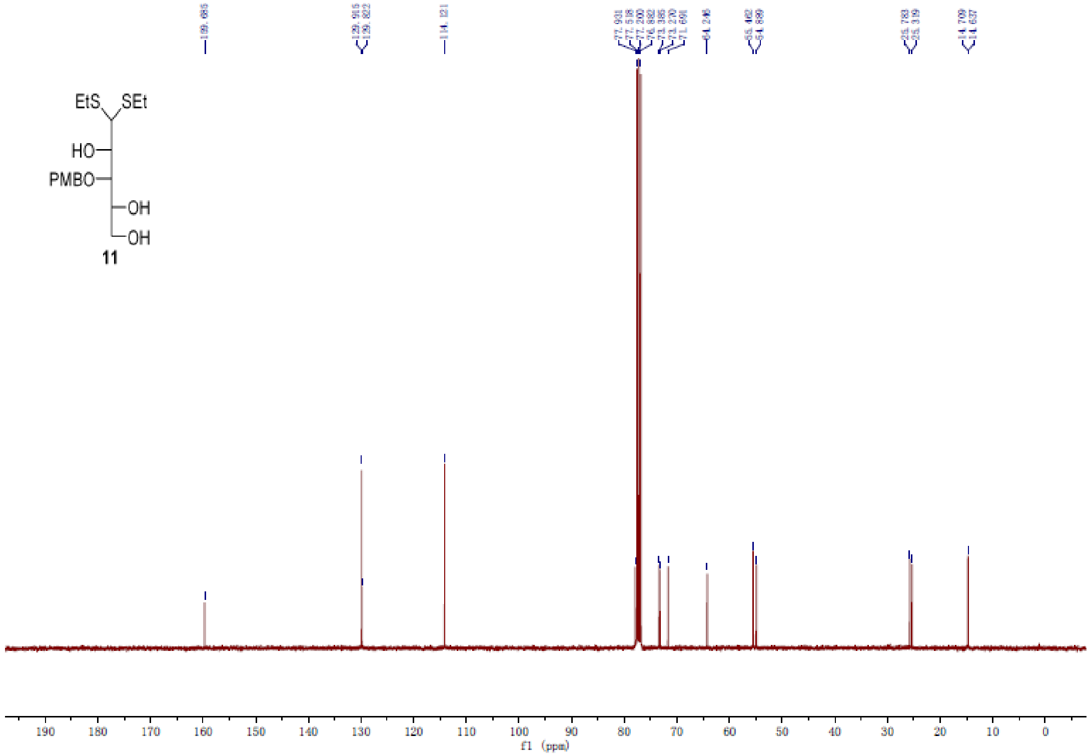

CDCl$_3$, 500 MHz

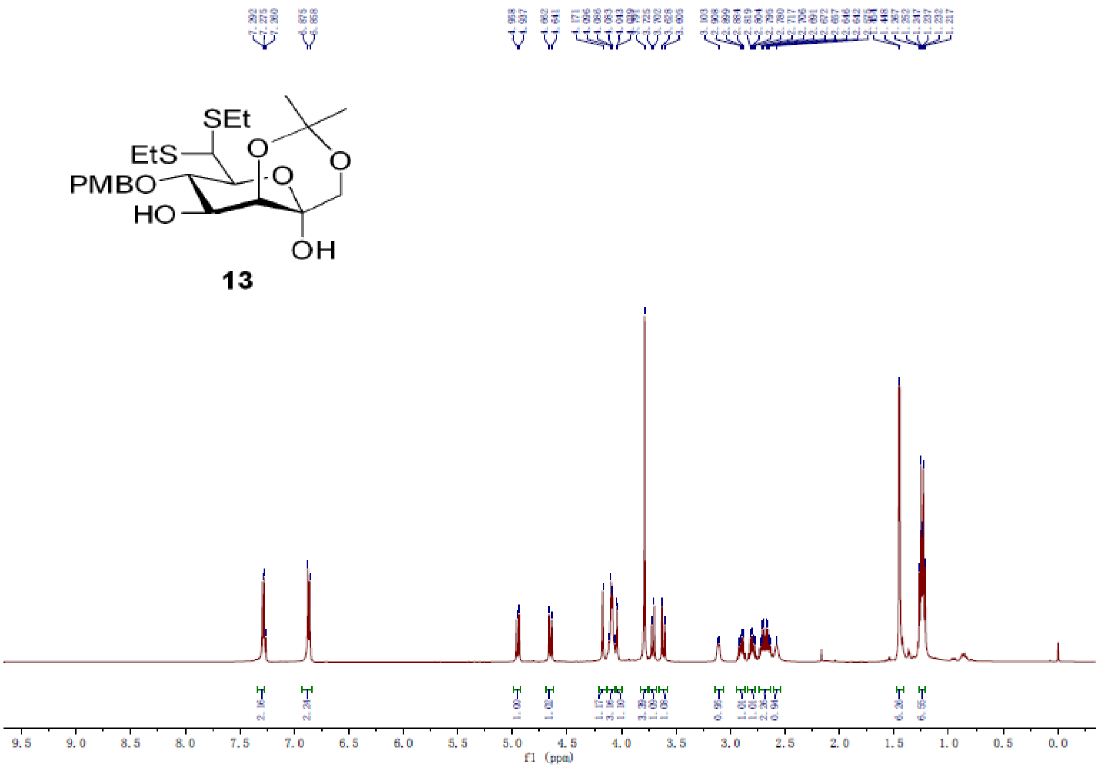

CDCl$_3$, 125 MHz

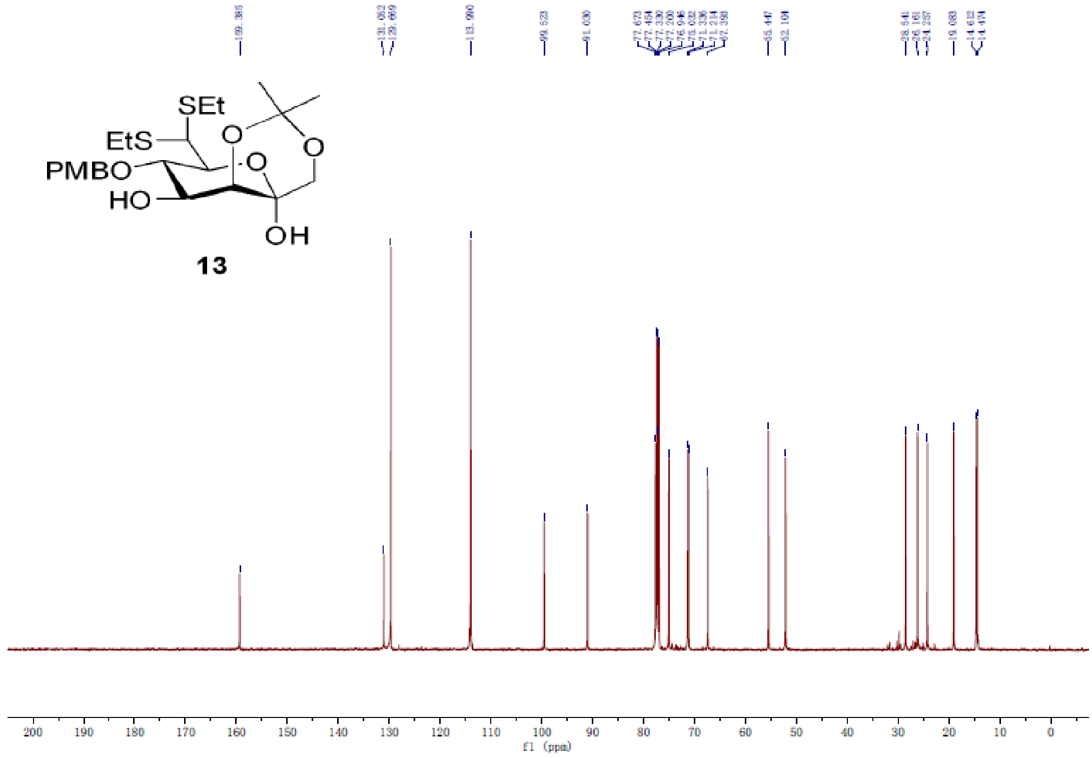

$CDCl_3$, 500 MHz

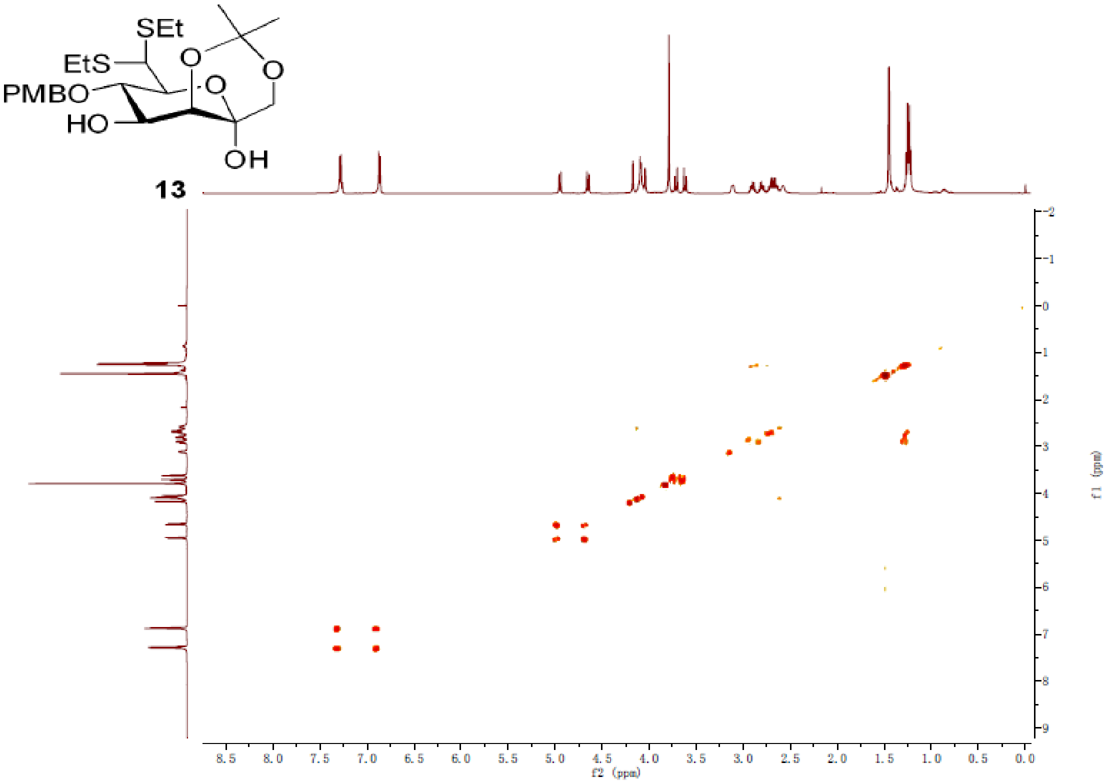

$CDCl_3$, 500 MHz

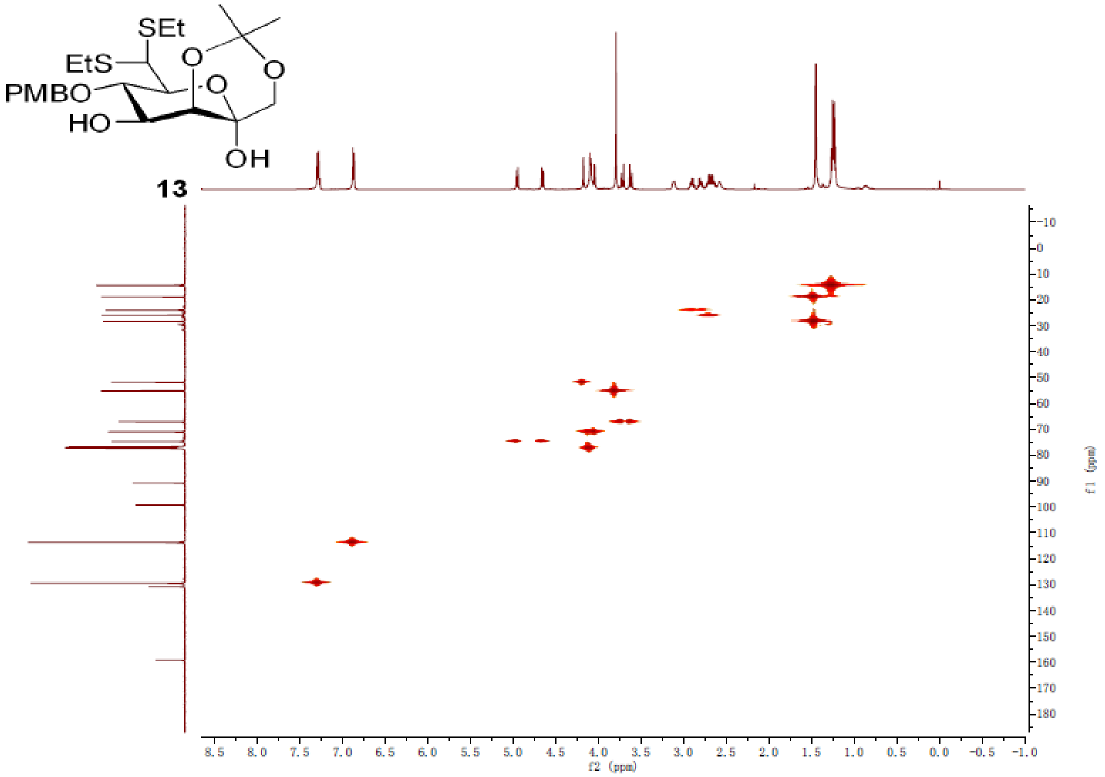

CDCl$_3$, 400 MHz

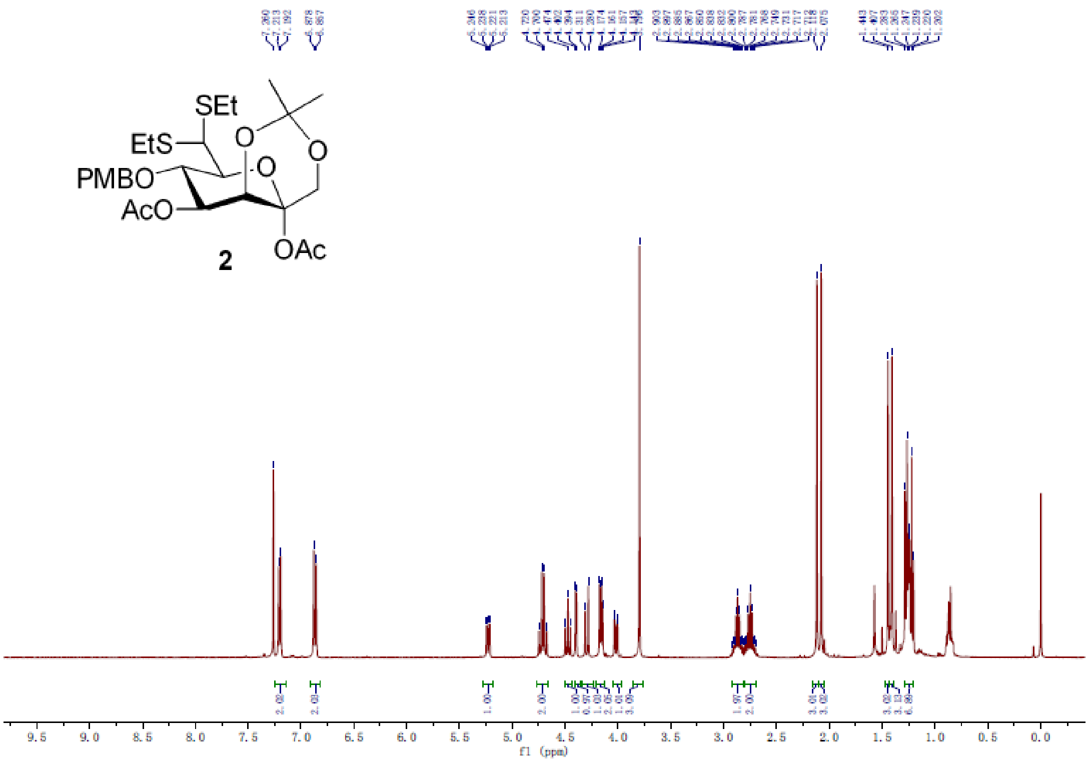

CDCl$_3$, 125 MHz

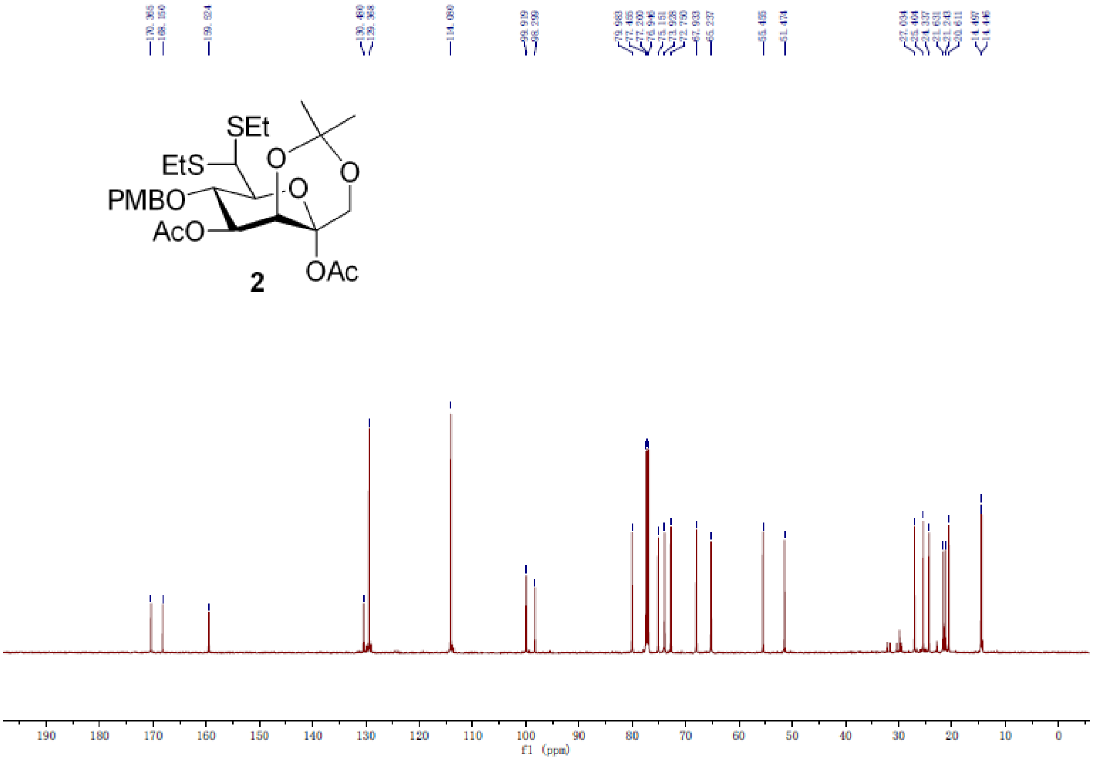

CDCl$_3$, 400 MHz

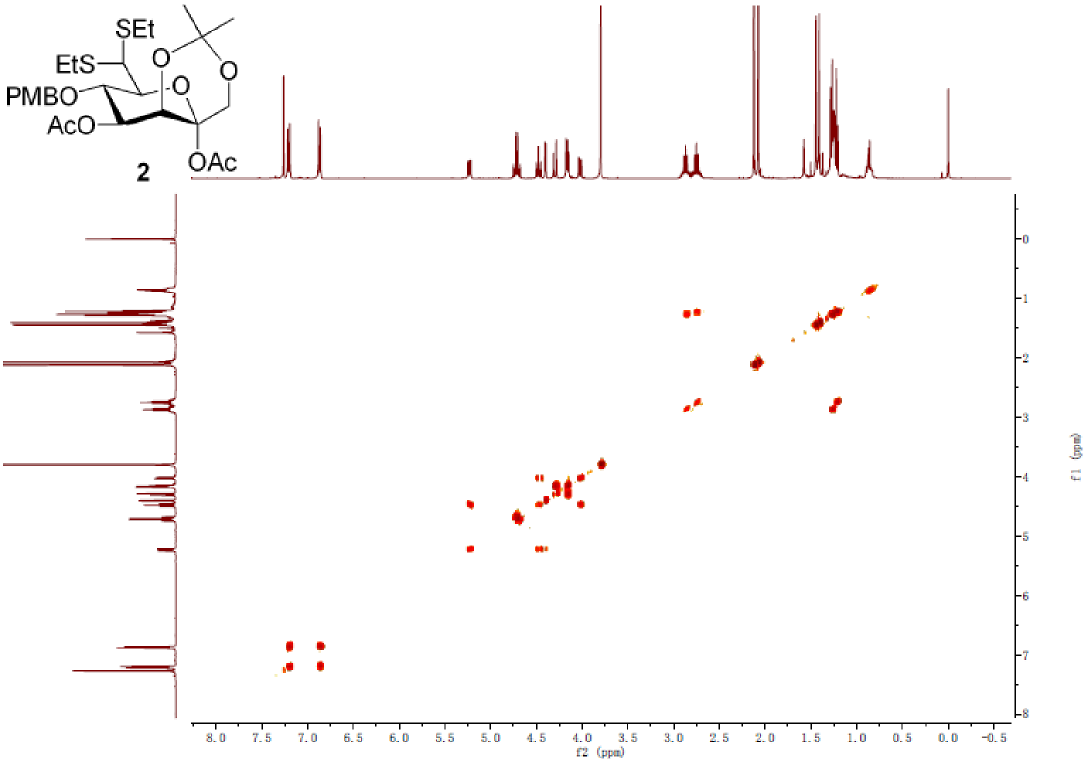

CDCl$_3$, 500 MHz

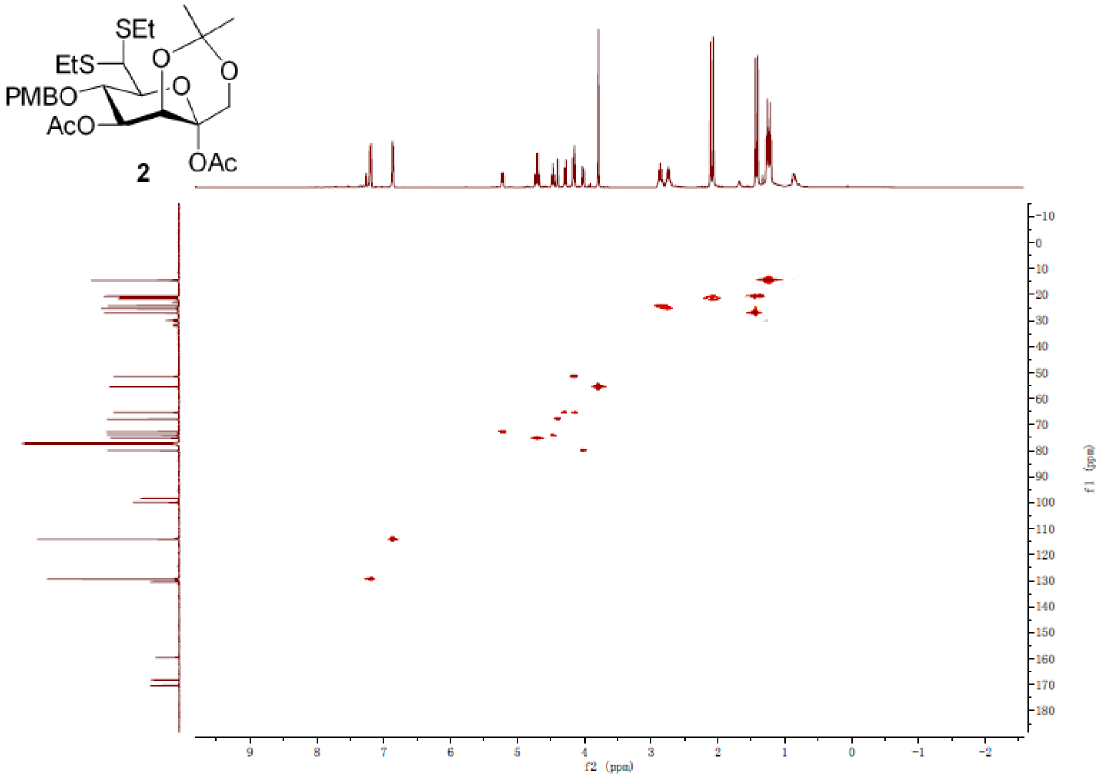

CDCl$_3$, 400 MHz

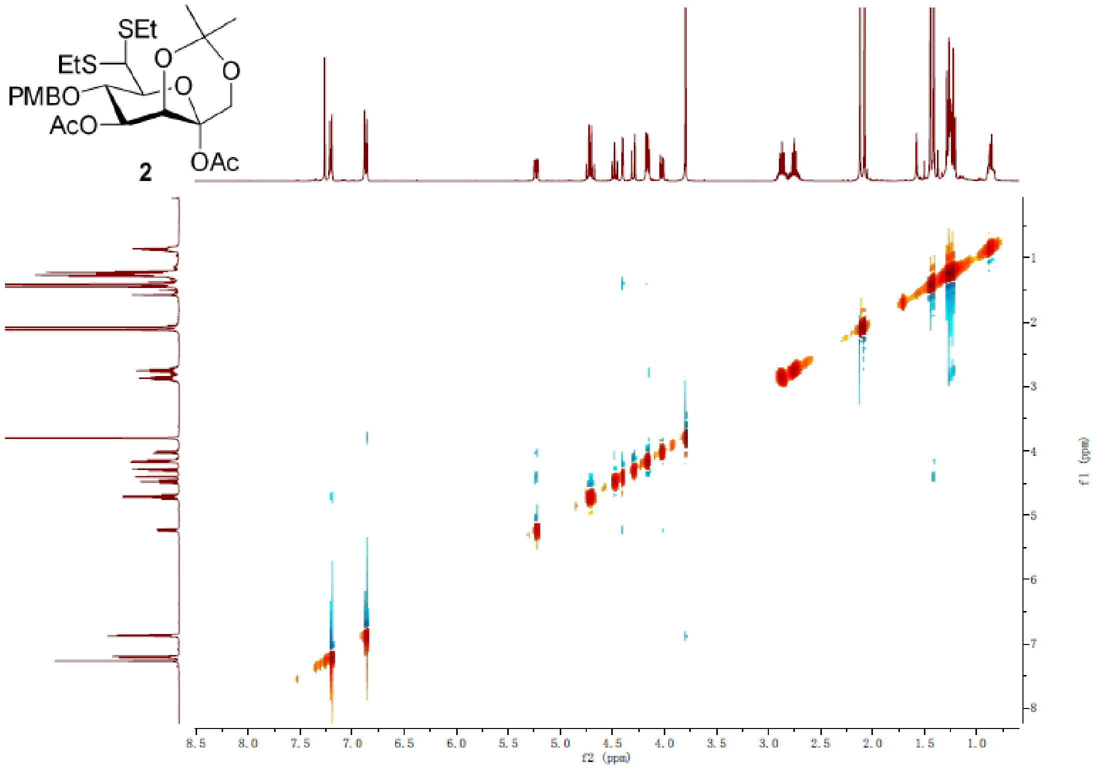

CDCl$_3$, 400 MHz

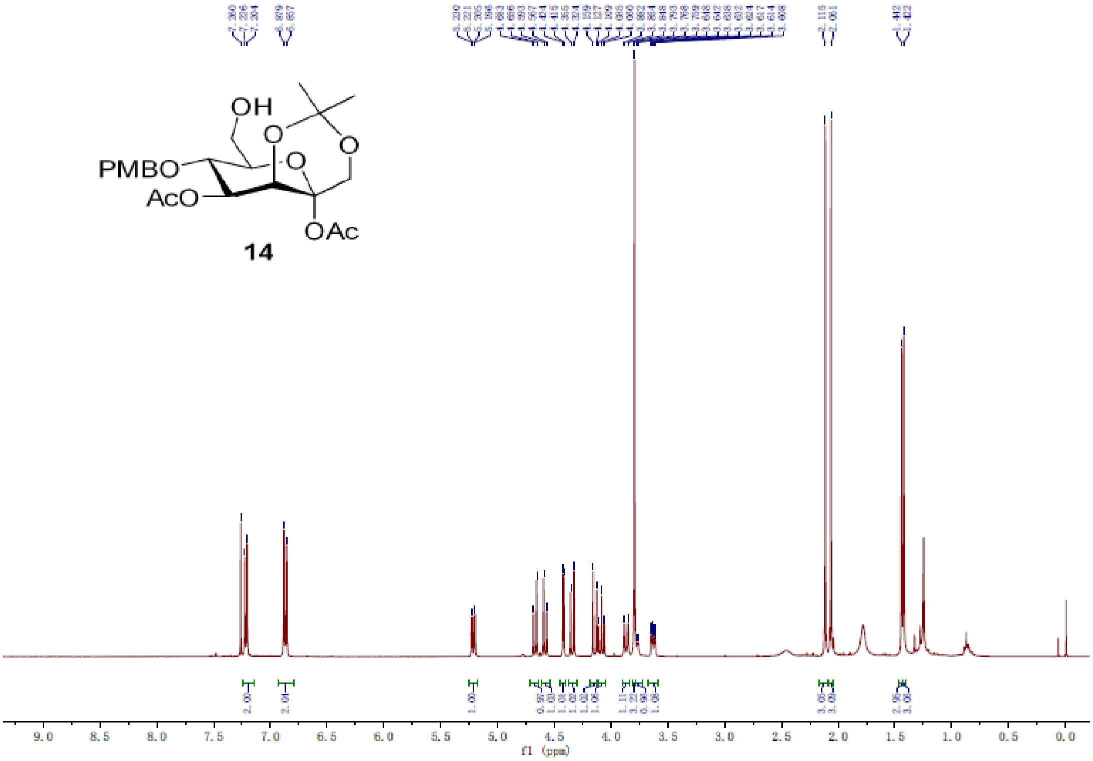

CDCl$_3$, 100 MHz

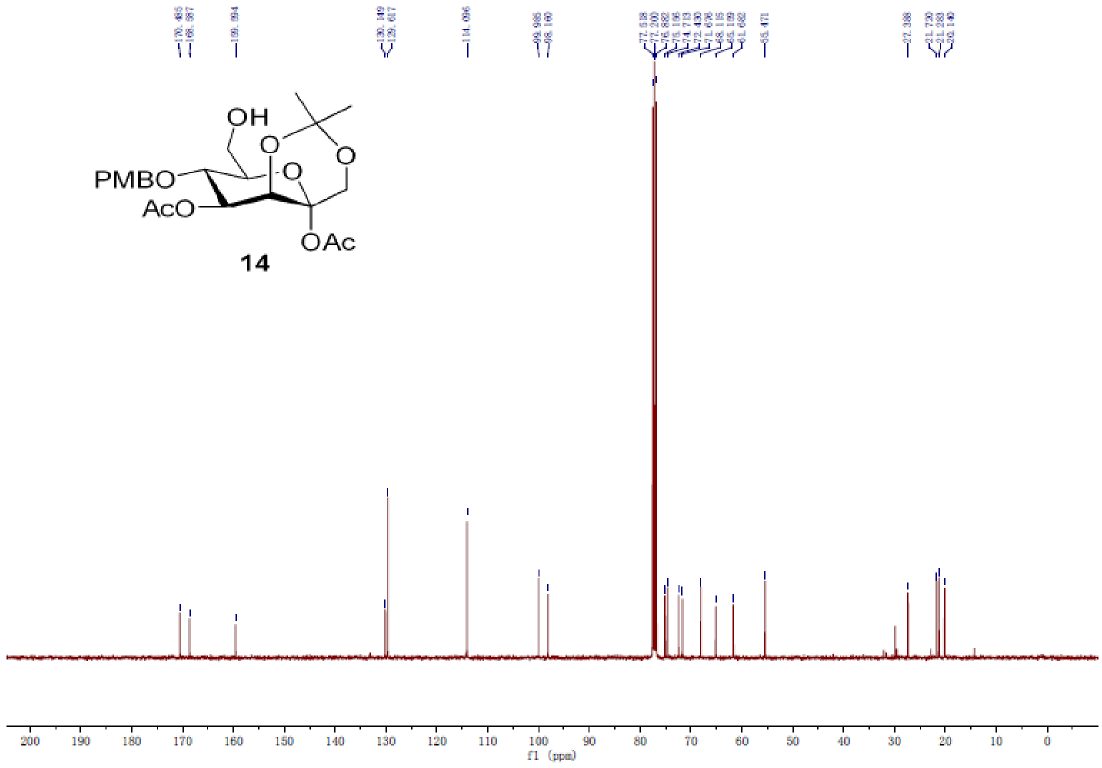

CDCl$_3$, 400 MHz

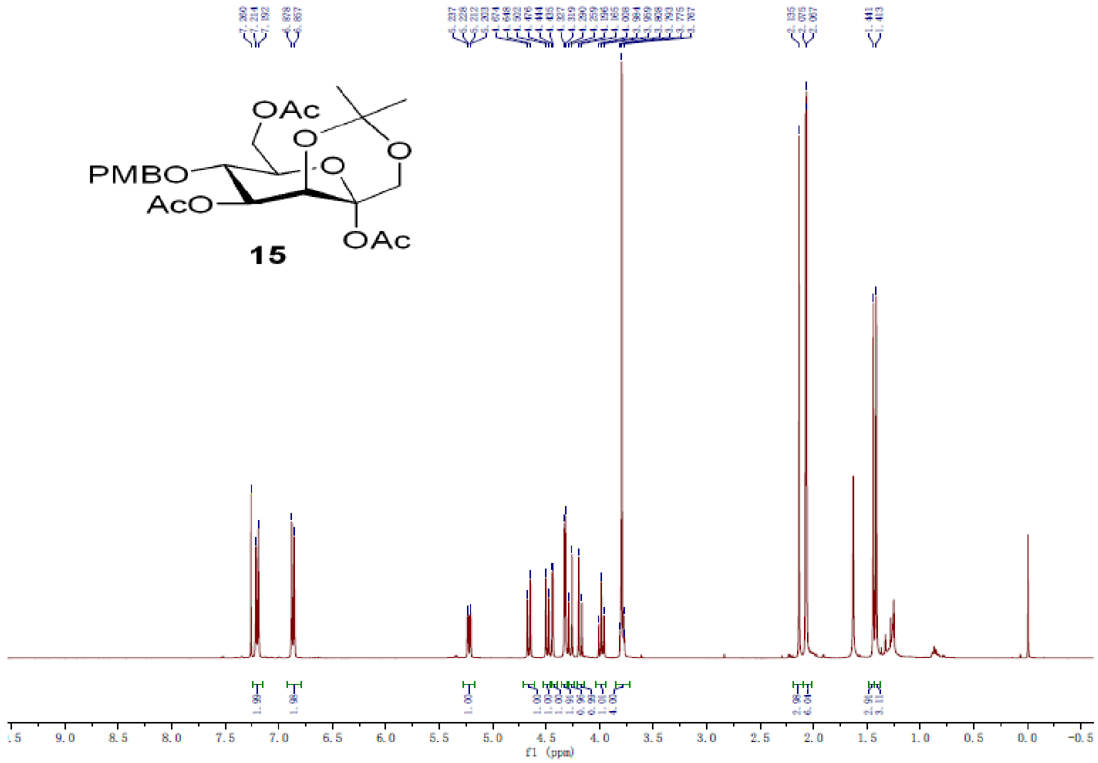

$CDCl_3$, 100 MHz

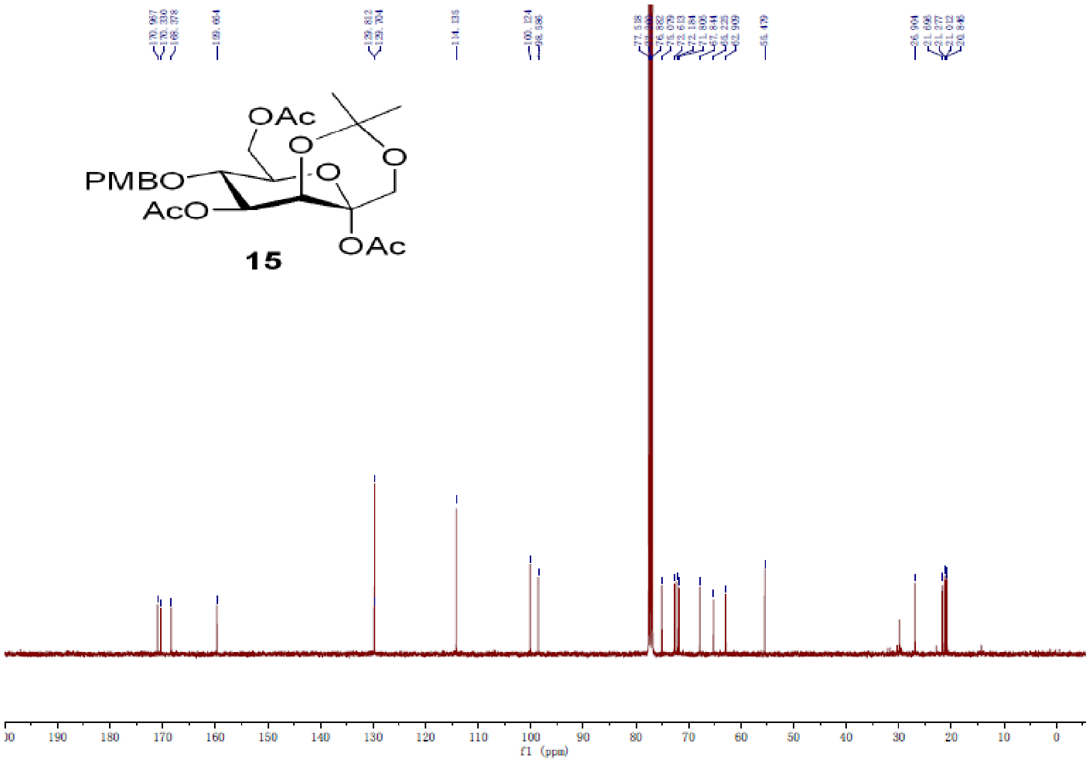

$CDCl_3$, 400 MHz

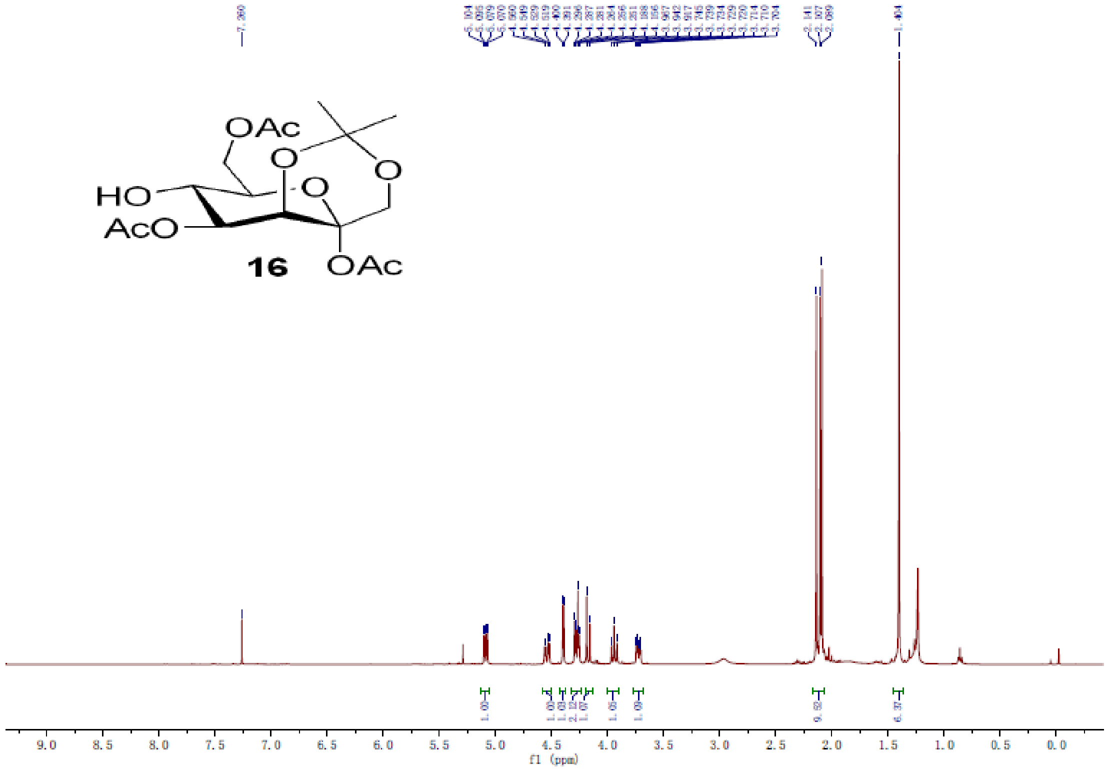

$CDCl_3$, 100 MHz

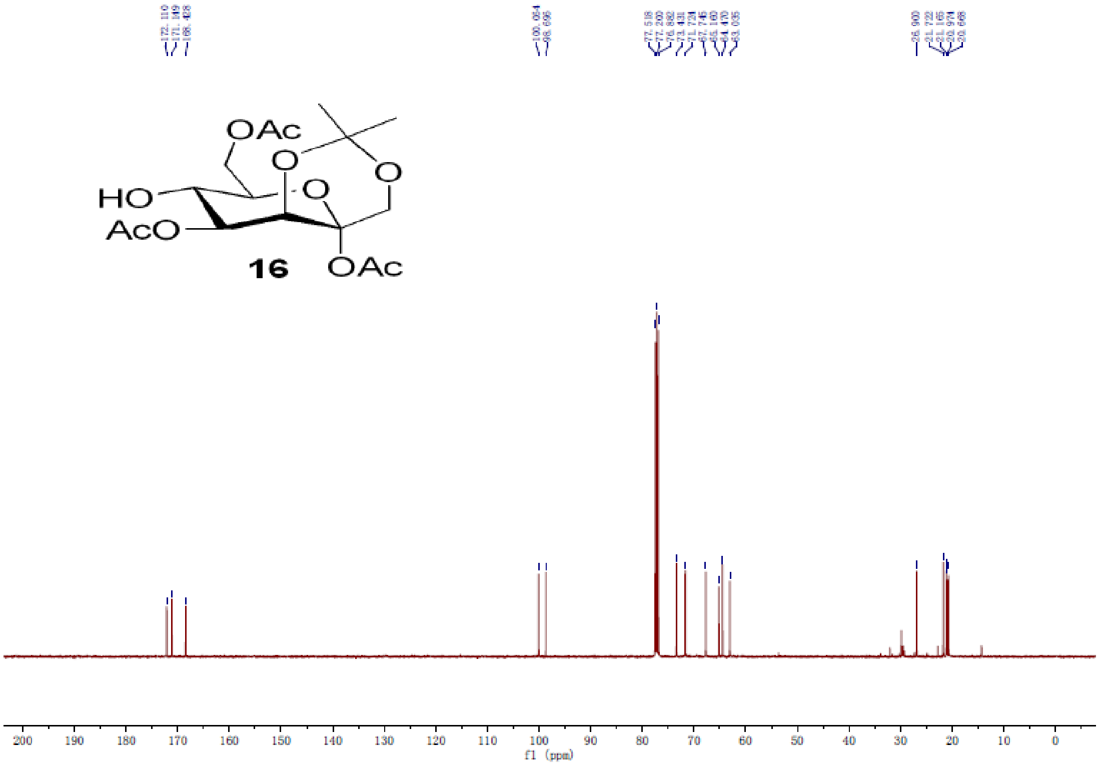

D_2O, 400 MHz

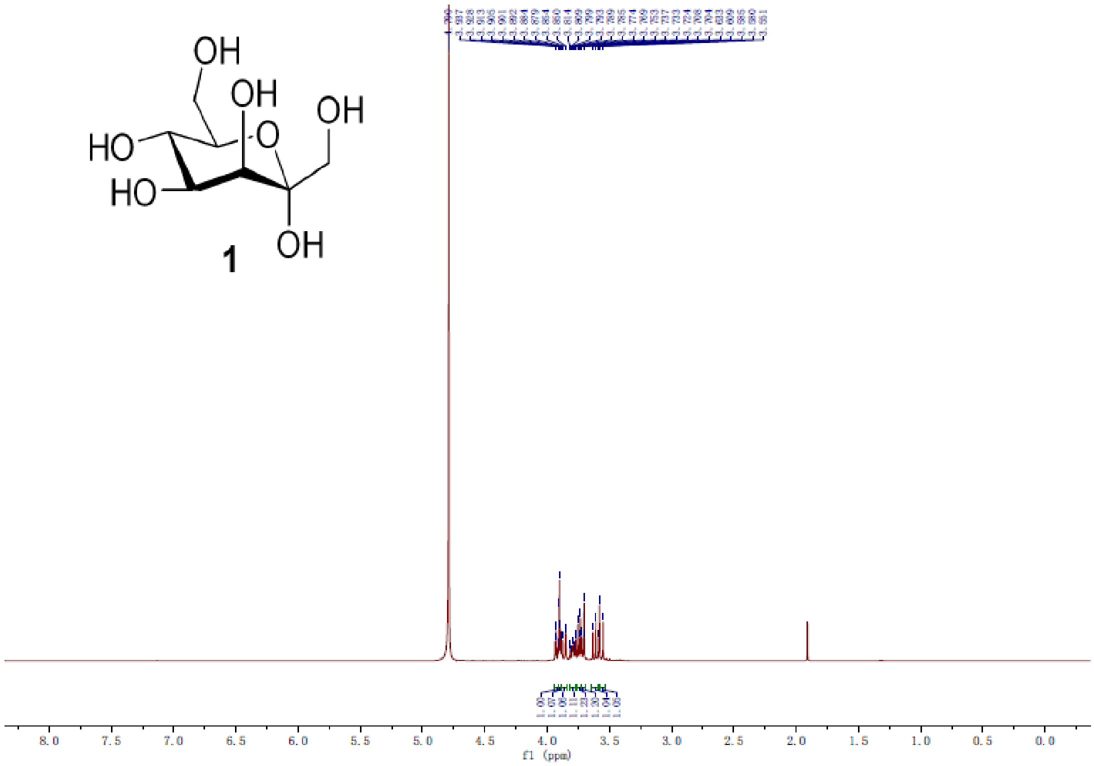

D$_2$O, 100 MHz

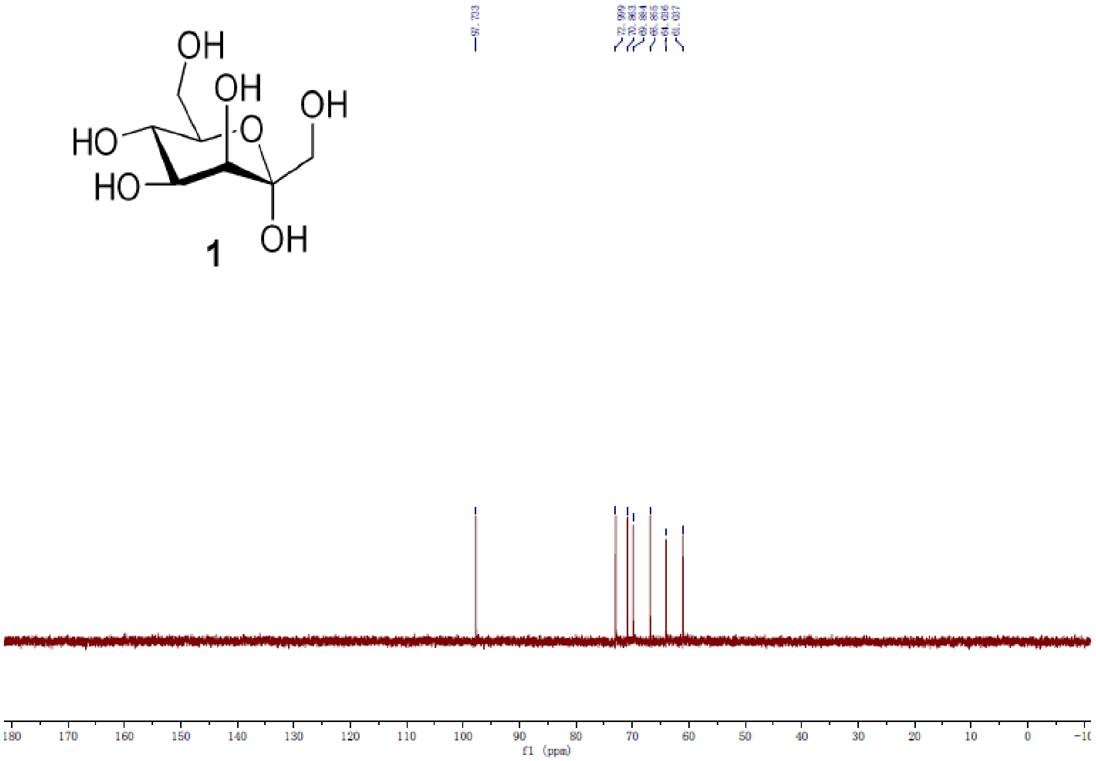
OH
OH
OH
HO
O
HO
OH
1
97.733
72.999
70.863
69.884
66.865
64.036
61.037
180
170
160
150
140
130
120
110
100
90
80
70
60
50
40
30
20
10
0
f1 (ppm)
